# Farmed Escapees Threaten MHC Diversity in Wild Atlantic Salmon

**DOI:** 10.1111/eva.70278

**Published:** 2026-05-29

**Authors:** Morten Lukacs, Åse Helen Garseth, Sten Karlsson, Kyrre Kausrud, Ottavia Benedicenti, Arvind Y. M. Sundaram, Lars Austbø, Cathrine Arnason Bøe, Håvard Lo, Unni Grimholt

**Affiliations:** ^1^ Norwegian Veterinary Institute Ås Norway; ^2^ Norwegian Institute for Nature Research Trondheim Norway; ^3^ Department of Medical Genetics Oslo University Hospital Oslo Norway

**Keywords:** Atlantic salmon, diversity, farmed salmon, Illumina, MHC, wild salmon

## Abstract

Wild Atlantic salmon populations in Norway and elsewhere are experiencing long‐term declines driven by reduced marine survival, climate change, impacts from aquaculture, introduction of alien species, and environmental degradation. Understanding how immune diversity is affected by these declines, and thus the ability of Atlantic salmon to combat current and future invading pathogens, is essential for the success of conservation programs such as gene banks. Major histocompatibility complex (MHC) genes, some of the most polymorphic genes known to date, are essential in the host immune defense against invading pathogens. Here we investigate MHC class I and class II diversity in escaped farmed salmon and in eight wild Atlantic salmon populations defined as being at risk. The study demonstrates that rare alleles can be found in single closely related wild populations within a fjord, and collectively, the populations hold a large immune diversity. Moreover, our study found that a broad spatial and temporal collection of escaped farmed salmon had an MHC‐diversity at the same level as individual wild salmon populations, while escaped farmed salmon from single escape events had lower diversity. Overall, the wild salmon had considerably higher MHC diversity than escaped farmed salmon. Accumulated genetic introgression of escaped farmed salmon is therefore a major threat as it can compromise the MHC diversity within and among the wild populations. Conservation of MHC diversity in the ongoing gene bank program for Norwegian Atlantic salmon is important, and our observation emphasizes that a large number of founders to the gene bank are needed to conserve the many rare MHC alleles and that genotyping of potential founders may be useful in broodfish selection.

## 1 Introduction

Atlantic salmon ( *Salmo salar* ) is a diadromous fish species that with high accuracy return to the river of birth after long‐distance feeding migration in the marine environment (Rikardsen et al. 2021; Thorstad et al. 2010). This homing behaviour has led to the structuring of the species into more than 2000 genetically distinct populations (Verspoor et al. 2005), of which approximately 450 are distributed in Norwegian rivers. Some river systems support a single distinct population, others contain multiple populations, and some rivers form metapopulations (Verspoor et al. 2005). In Norway, Atlantic salmon are managed at the population level to ensure the maintenance of genetic distinctiveness, and at the same time to ensure population sizes large enough to maintain genetic diversity (Norwegian Ministry of the Environment 2007; Hindar et al. 2010).

After long‐term decline, and despite comprehensive management efforts, wild Atlantic salmon were assigned to the *near threatened* category on the Norwegian Red list for species in 2021 and the IUCN Red List of Threatened Species in 2023 (Darwell 2023; Hesthagen et al. 2021). The annual number of wild Atlantic salmon returning from the ocean is now one‐third of the level recorded in the 1980s (Scientific Advisory Committee for Atlantic Salmon Management 2025; Thorstad et al. 2021). The main reasons for the decline are large‐scale reduction in marine survival and anthropogenic factors such as aquaculture, environmental degradation, habitat fragmentation, introduction of alien species and climate change (Scientific Advisory Committee for Atlantic Salmon Management 2025; Forseth et al. 2017). Loss of genetic integrity due to introgression of escaped farmed Atlantic salmon (Karlsson et al. 2016) and mortality in migrating post‐smolt due to infestation of salmon lice (*Lepeoptheirus salmonis*) from aquaculture are two well‐documented threats (Forseth et al. 2017; Glover et al. 2017; Thorstad et al. 2021; Vollset 2015). On the other hand, knowledge is scarce on the impact of increased infection pressure due to disease outbreaks in farmed Atlantic salmon and rainbow trout in open net pens along the coast (Scientific Advisory Committee for Atlantic Salmon Management 2025; Johansen et al. 2011; Taranger et al. 2015).

Climate change adds layers of challenges to the species by increasing sea surface temperatures and affecting both the fitness and availability of food. Expected shifts in the geographic distribution of fish communities and alterations of microbial fauna might expose Atlantic salmon to new pathogens (Scientific Advisory Committee for Atlantic Salmon Management 2021; Cheung et al. 2015; Fossheim et al. 2015; Hoegh‐Guldberg and Bruno 2010; Straneo and Heimbach 2013; Thorstad et al. 2021). Thus, under climate change, immunological diversity becomes increasingly important, with large and diverse populations having a greater capacity for adaptation.

Certain molecules on the surface of cells, called MHC class I and class II, help the immune system recognize threats. They work by showing small pieces of proteins (antigens) to immune cells called T cells, which triggers an immune response. MHC class I molecules display proteins from inside the cell, such as those from viruses, to CD8^+^ “killer” T cells, which can destroy infected cells. MHC class II molecules display proteins that come from outside the cell, such as bacteria, to CD4^+^ “helper” T cells, which then activate antibody‐producing immune responses (Buso et al. 2025).

In Atlantic salmon, there is only one classical MHCI gene, denoted *UBA*, and single classical MHCII alpha and beta genes, denoted *DAA* and *DAB* (reviewed in (Grimholt 2016)). The *UBA* locus resides on chromosome 27 while the *DAA* and *DAB* loci reside closely linked on chromosome 12 and segregate as a *DAA*‐*DAB* haplotype. All three genes are polymorphic, with 48, 22, and 42 alleles from the *UBA*, *DAA*, and *DAB* loci currently registered in the IPD‐MHC database (https://www.ebi.ac.uk/ipd/mhc/group/FISH/, (Ballingall et al. 2018)). These sequences originate from various studies on Atlantic salmon from the Northern Hemisphere.

Being unlinked, the *UBA* and *DAA*‐*DAB* genes seem to be subjected to different selective pressures (Consuegra, Megens, Leon, et al. 2005; Consuegra, Megens, Schaschl, et al. 2005). Sequence identity between MHCII alleles is much higher than that found for MHCI alleles. Some of this added MHCI diversity is due to recombination occurring in the large intron between the *UBA* alpha 1 to alpha 2 domains which exchanges alpha 1 domain sequences between different alpha 2 and downstream sequences (Aoyagi et al. 2002; Grimholt et al. 2015; Kiryu et al. 2005).

In a previous study, we investigated MHC diversity in 81 Atlantic salmon from one of four Norwegian breeding core populations (Gjedrem et al. 1991; Gjøen and Bentsen 1997) i.e., the top level of the breeding pyramid, where genetic selection and improvement take place, and found eleven *UBA* alleles and seven *DAA*‐*DAB* haplotypes (Grimholt et al. 2003), suggesting a medium level of diversity. However, a more recent pilot study of only nine Atlantic salmon from an endangered Norwegian population displayed twelve MHCI alleles and eight MHCII haplotypes, where one *DAB* and six *UBA* alleles were not present in the IPD‐MHC database (Sundaram et al. 2020). This suggested that the MHC diversity might be much larger in wild salmon than what we previously found in farmed salmon (https://www.ebi.ac.uk/ipd/imgt/hla/about/statistics/). The MHC diversity in wild and in farmed Atlantic salmon in Norway is to our knowledge not monitored and thus largely unknown. Knowledge about MHC diversity is a valuable tool also in preservation and restoration of populations at risk, such as those included in the live gene bank program from the Hardanger area. To understand how long‐term increase in infection pressure, genetic introgression, climate change and other environmental factors have affected this MHC diversity, we set out to quantify the MHC diversity in wild salmon from eight populations defined as at‐risk within the Hardanger region. We compared this diversity against Atlantic salmon of defined farmed origin.

## 2 Materials and Methods

### 2.1 Study Material

**FIGURE 1:**
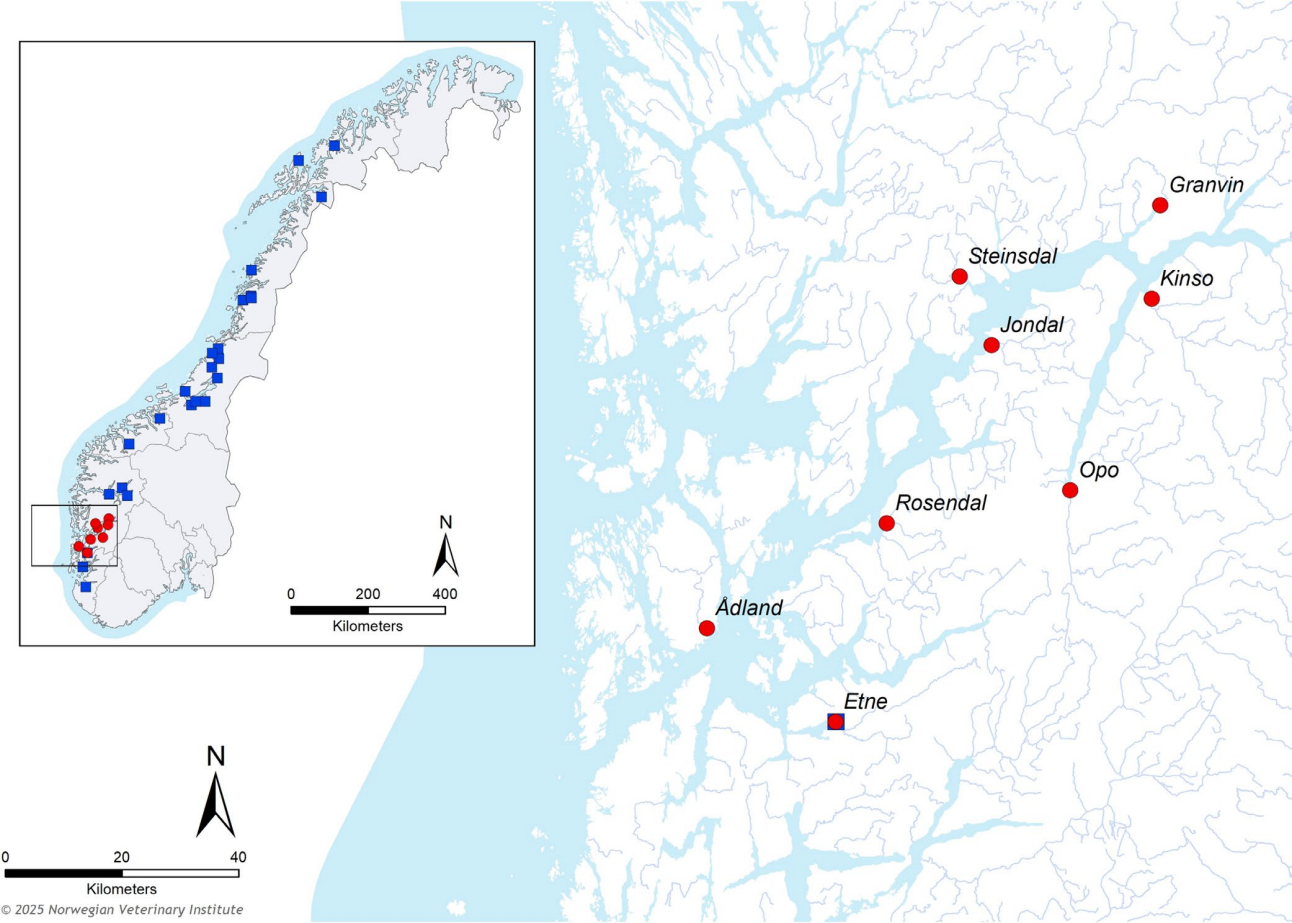
Map of Atlantic salmon sampling sites in Norway. Sampling sites for farmed escapees are shown using blue squares while the Hardanger fjord area is enlarged and wild salmon sampled in river populations are shown using red circles. Both wild and farmed escapes are sampled from the Etne river, here shown using both a red circle and a blue square. Additional data on wild salmon and farmed escapes are shown in Appendix S1.

The study material comprised wild Atlantic salmon captured in the following eight rivers in the Hardanger region of Western Norway: Etne, Granvin, Jondal, Kinso, Opo, Rosendal, Steinsdal, and Ådland (Figure 1, Appendix S1). All eight wild river populations are defined as being in a poor or very poor state (Norwegian Ministry of Climate and Environment 2013; Scientific Advisory Committee for Atlantic Salmon Management 2021; Forseth et al. 2013) (Appendix S1.01).

All head‐kidney and spleen tissues used in this study were sampled during routine post‐mortem health examination of wild salmon that served as founder fish when the live gene bank was established in Hardanger (Gausen 1993). All founders had to pass this health check and, in addition, be approved as wild in a two‐step investigation of genetic integrity which comprises a visual analysis of the growth pattern of scales followed by genetic analyses. Only salmon approved according to the criteria defined for the gene bank program are denoted as wild salmon in this study.

After capture, all potential broodfish for live gene bank are tagged (Floy‐tags) and scales are sampled dorsal of the lateral line, posterior of the dorsal fin, deposited in special scale sample envelopes where Floy‐tag number, species, river of origin, date of capture, sex, weight and body length are recorded. The scales are then scanned and photographed by a microscope, and the growth pattern is inspected manually. Salmon that exhibit a scale pattern consistent with a farmed origin (Fiske et al. 2005; Lund and Hansen 1991) are excluded as founder fish in the gene bank program at this first step. Because genetic introgression of escaped farmed salmon is widespread in Norway (Diserud et al. 2022; Karlsson et al. 2016), salmon that have a growth pattern consistent with a natural life history are further analyzed for potential genetic introgression of escaped farmed salmon. From a set of 48 SNP‐markers that generically differentiate between wild and farmed salmon (Karlsson et al. 2011) the probability of being of wild (P(wild)) versus farmed origin is estimated by applying the statistical method by Karlsson et al. (2014). Individuals with a *p* (wild) < 0.71 are excluded as founder fish in the gene bank.

The health check comprises post‐mortem examination by authorised fish health personnel, including sampling of target tissues for qPCR analyses for vertically transmitted pathogens (Boe et al. 2021; Garseth et al. 2013). Tissue samples (gill, myocardium, head kidney, and spleen) are preserved in RNAlater. Broodfish are tested with PCR analyses for vertically transmitted pathogens including *Renibacterium salmoninarum* and infectious pancreatic necrosis virus (IPNV). In addition, the health monitoring program includes piscine orthoreovirus‐1 (PRV‐1), piscine myocarditis virus (PMCV), and infectious salmon anaemia virus (ISAV) where the knowledge regarding vertical transmission is uncertain. *Renibacterium salmoninarum* , IPNV, and ISAV were not detected in any of the wild salmon included here. PMCV was detected in altogether eight broodfish from Rosendal (2), Ådland (1), Kinso (1), and Opo (4). PRV‐1 occurred in a low proportion of salmon from most rivers. Fish with PMCV and PRV‐1 were excluded as founder fish but not omitted from the dataset in the present analyses.

After the qPCR analyses, residuals are stored in the gene banks biorepository (NGB biobank) for retrospective analyses and research. For this study, samples of spleen or head kidney from 265 wild Atlantic salmon sampled from 2016 to 2019, originating from eight rivers in the Hardanger region, were donated by the gene bank biorepository. The number of samples included from each river and time of sampling are shown in Table 1 with expanded details in Figure 1 and Appendix S1.

**TABLE 1:** Overview of wild and escaped farmed Atlantic salmon (*N*_ind_) assayed for genetic variation in MHC class I and class II, and 70 SNPs.

Population	Sampling period	*N*_ind_
Granvin	2017–2019	22
Jondal	2016–2019	17
Kinso	2016–2019	21
Rosendal	2016–2019	40
Ådland	2016–2019	45
Etne	2017–2018	41
Opo	2016–2019	52
Steinsdal	2016–2019	27
Escapees	2007–2020^a^	90
Total		355

^a^ See Figure 1 and Appendix S1 for details.

To evaluate the potential impact of genetic introgression from escaped farmed salmon on the MHC diversity in the wild Atlantic salmon, we also analyzed samples from 90 salmon identified as escaped farmed salmon based on the growth pattern of scales (Fiske et al. 2005; Lund and Hansen 1991). 79 of these individuals were caught in 17 different rivers along the Norwegian coast during 2007–2010, ranging from one to 34 individuals per river, where the 34 escapees from Etne (2010) were reported to come from a single escape event (Figure 1, Appendix S1.02). An additional 11 escaped salmon were caught at sea in five different production areas (PA 2, 6, 7, 8, and 10; Norwegian Ministry of Trade, Industry and Fisheries 2017), during 2019 and 2020 (Garseth 2021).

### 2.2 Preparing the Sequence Library

Spleen or head kidney samples preserved in RNAlater (Thermo Fischer Scientific, Waltham, USA) from the NGB biobank were dissolved using Tissue lyzer (Qiagen, Hilden, Germany) with MagNA Pure LC RNA Isolation Tissue Lysis Buffer (Roche Molecular Systems, Mannheim, Germany). RNA was isolated using MagNA Pure 96 Cellular RNA Large volume kit (Roche Molecular Systems) according to the manufacturer's recommendation. RNA integrity and concentrations were measured on TapeStation capillary electrophoresis (Agilent, Santa Clara, CA, USA) and Qubit fluorometer (Invitrogen, Carlsbad, CA, USA). cDNA was synthesized using up to 1000 ng total RNA and the QuantiTect Reverse Transcription Kit (Qiagen) according to the manufacturer's recommendation, resulting in 20 μL cDNA stored at −20°C for later use.

Based on a priori knowledge, we used three forward primers to capture *UBA* alleles paired with one reverse primer (Grimholt et al. 2015; Kiryu et al. 2005; Sundaram et al. 2020). The UBA.F1 primer amplifies alleles from the alpha 1 domain lineages I, II, V, and VI, the UBA.F2 primer amplifies those from lineage III and VII and the UBA.F3 primer amplifies alleles from lineage IV. For *DAA* and *DAB* we used single forward and reverse primers, which have previously been shown to amplify all alleles. Primer sequences are shown in Appendix S1.1.

We used 10 ng of the cDNA in 20 μL PCR reactions for each PCR reaction with KAPA HiFi HotStart ReadyMix (Roche Molecular Systems) and 0.1 μM each primer (Appendix S1.1). The following program was used for amplification: 94°C for 2 min; 25 cycles of 94°C for 30 s, 58°C for 30 s, 72°C for 60 s; 72°C for 10 min. Products were verified on a 1% agarose gel prior to clean up using 1.8 × PCR volume of Agencourt AMPure XP PCR purification kit (Beckman Coulter, Brea, CA, USA) according to the manufacturer's recommendation and dissolved in 30 μL TE. DNA concentrations and fragment sizes were measured on a Qubit fluorometer (Invitrogen, Carlsbad, CA, USA) and Agilent Bioanalyzer (Santa Clara, CA, USA), respectively.

Libraries containing each of the *UBA*, *DAA*, and *DAB* PCR products were mixed in proportions for each individual to ensure similar coverage and subjected to 10 cycles of PCR using the second set of primers, thus adding two unique Illumina indexes per individual (Appendix S1.1). This second amplification was also carried out with the Kapa HiFi Hot Start PCR Kit as previously described. The following program was used for amplification: 94°C for 2 min; ten cycles of 94°C for 30 s, 58°C for 30 s, 72°C for 60 s; 72°C for 10 min. Amplicons were purified using 1.0 × PCR volume of Agencourt AMPure XP PCR purification kit (Beckman Coulter) according to manufacturer's recommendation, and dissolved in 30 μL TE. All samples were finally pooled together and concentrated with 1.0 × PCR volume of AMPure XP beads. Based on data from Tapestation and Qubit, the PCR pool was mixed, totaling 2 μg DNA and sequencing was performed on Illumina MiSeq platform (Illumina, San Diego, CA, USA) using the v3 chemistry to achieve 300 bp paired‐end reads.

### 2.3 Bioinformatics

#### 2.3.1 Sequence Analyses

Sequence data was demultiplexed using the Illumina indices introduced during the second PCR reaction resulting in fastq data (read 1 and read 2) for each individual. Fastq data has been submitted to NCBI SRA under the BioProject accession numbers PRJNA578031 and PRJNA1097785. NCBI accession numbers for MHC alleles not yet registered in the IPD‐MHC database are OL441523‐91 and OR667818‐26. The bioinformatic pipeline used in this study has been described in detail in Sundaram et al. (2020).

Raw data from each individual was processed using BBDuk v34.56 (part of BBTools; https://archive.jgi.doe.gov/data‐and‐tools/software‐tools/bbtools) to remove/trim bad quality reads and sequencing adapter sequences. Cleaned reads were further demultiplexed based on the primers used during the first PCR reaction using demultiplexer v1.7 (https://github.com/nsc‐norway/triple_index‐demultiplexing), allowing zero mismatches between the primers and the sequenced reads. This step separates the reads into each MHC subgroup as targeted during the first PCR reaction and removes the primer sequences from the reads.

Overlapping read 1 and read 2 for each MHC subgroup were combined using FLASH v1.2.11 (Magoc and Salzberg 2011) with default settings (−r 300 was used to specify the read length). The resulting full‐length amplified reads were collapsed to identify all the unique reads and sorted based on the number of times it was present in the data using fastx_collapser (part of FASTX Toolkit v0.0.13; http://hannonlab.cshl.edu/fastx_toolkit/). The top five most represented (1%–2% of the FLASHed reads) full‐length amplified reads (FASTA format) were analysed further.

Potential MHC allele sequences identified using the above approach were evaluated using custom Python scripts (https://github.com/NorwegianVeterinaryInstitute/Salmonid_MHC_classifier). During this step, 0–2 nucleotides were removed from both ends of the sequences to accommodate frame shift issues while converting to amino acid sequences before proceeding with the analyses. The scripts use a library of official IPD‐MHC Database alleles [IPD‐MHC Database (https://www.ebi.ac.uk/ipd/mhc/)] to identify the closer match to the input sequence. New alleles were added manually to a local database for verification purposes.

The input FASTA records were converted to amino acid sequences using transeq [part of EMBOSS v6.6.0.0; (Rice et al. 2000)] followed by multiple sequence alignment (only nucleotide) with relevant IPD‐MHC Database entries using MUSCLE v3.8.1551 (Edgar 2004). Closest clade/sibling information from the tree produced by MUSCLE was extracted using the python module ETE toolkit (Huerta‐Cepas et al. 2016). Sequence similarity and identity between the FASTA record and the closest sibling were calculated using Water [part of EMBOSS v6.6.0.0] alignment tool for both nucleotide and amino acid sequences, respectively. A report file was generated with all the relevant information for each FASTA record, which was subjected to further manual inspection and sequence phylogenies prior to allele designation.

New alleles are named in accordance with standing nomenclature (Ballingall et al. 2018) as follows: *UBA* allele sequences differing from previous alleles in more than 4 amino acids are defined as a new two‐digit allele, while *DAA* and *DAB* alleles must differ from existing alleles by more than three amino acid sequences to entitle a new two‐digit allele name. Sequences differing by less than four amino acids for *UBA* or three amino acids for *DAA* and *DAB* are given a new four‐digit number, as exemplified by, for instance DAA*01:01 and DAA*01:02. Alleles with nucleotide differences not resulting in amino acid changes are given an additional six‐digit extension, exemplified by for instance DAA*03:03:01 or DAA*03:03:02.

#### 2.3.2 SNP‐Genotyping

The wild salmon were also assayed for genetic variation at loci across the genome, including 68 putatively neutral SNPs and the Vgll3 and the Six‐6 loci tightly associated with age at maturity in Atlantic salmon (Barson et al. 2015). The putatively neutral SNPs are SNPs shown to generically differentiate between wild and farmed salmon (Karlsson et al. 2011), of which 48 are being used for monitoring farmed‐to‐wild genetic introgression (Karlsson et al. 2016, 2014). DNA was extracted from scale samples using a semiautomatic King Fisher Apex System extraction robot and the MagMax DNA Multi‐Sample Ultra 2.0 kit. The genotyping was conducted on an EP1 96.96 Dynamic array IFCs, Fluidigm platform.

#### 2.3.3 Phylogenetic Analysis

The evolutionary history was inferred by using the Maximum Likelihood method based on the Jukes‐Cantor model (Jukes and Cantor 1969). Initial tree(s) for the heuristic search were obtained automatically by applying Neighbor‐Joining and BioNJ algorithms to a matrix of pairwise distances estimated using the Maximum Composite Likelihood (MCL) approach and then selecting the topology with superior log likelihood value. All positions with less than 95% site coverage were eliminated. That is, fewer than 5% alignment gaps, missing data, and ambiguous bases were allowed at any position. Evolutionary analyses were conducted in MEGA7 (Kumar et al. 2016).

#### 2.3.4 Genetic Analysis

Diversity statistics metrics were calculated for each population and each data type. For MHC we focused on *UBA* and *DAB* as *DAA* alleles segregate in haplotypes with alleles from the closely linked *DAB* gene (Grimholt et al. 2003). Expected and Observed Heterozygosity were calculated using the summary function in the adegenet R package (Jombart and Ahmed 2011) while allelic richness, including rarefied allelic richness, for SNPs and MHC loci were calculated using the allelic.richness function in the hierfstat R package (Goudet 2005). To evaluate genetic differentiation among populations, the fixation index (*F*_ST_) was calculated for SNPs and MHC loci using the genet.dist function within the adegenet R package. As a non‐parametric alternative to the *t*‐test, the Wilcoxon rank‐sum test for independent samples was used for *F*_ST_ results.

#### 2.3.5 Statistical Analysis

Allele frequencies were calculated for samples from different rivers and sexes, as well as for escaped farmed salmon. Rarefaction analysis, as well as an estimation of allelic richness, was done according to the extrapolation method by (Foulley and Ollivier 2006) as implemented in R Pegas (Paradis 2010). Further simulation was conducted by estimating the empirical frequency distribution (Venables and Ripley 2002) of alleles from the full population and re‐sampling from hypothetical populations with increasing numbers of alleles present, matching the observed accumulation of unique alleles to these simulations. Finally, the Shannon‐Weaver diversity index was used as a metric of allelic diversity across levels (Oksanen et al. 2025; Sherwin 2018) and bootstrapped accumulation curves for diversity were made by 1 K iterations of leaving out 1/3 of the samples. Non‐parametric regression splines were fitted as part of generalized additive models addressing differences between rivers, sexes, and farmed versus wild‐type (Wood 2003, 2017), while parametric Michaelis–Menten accumulation functions were fitted through non‐linear least squares.

## 3 Results

We investigated MHC diversity in 265 wild Atlantic salmon originating from eight rivers in the Hardanger area and 90 escaped farmed salmon captured in rivers and fjords along the Norwegian coast (Figure 1).

### 3.1 *UBA* , the Single Classical MHC Class I Gene

**FIGURE 2:**
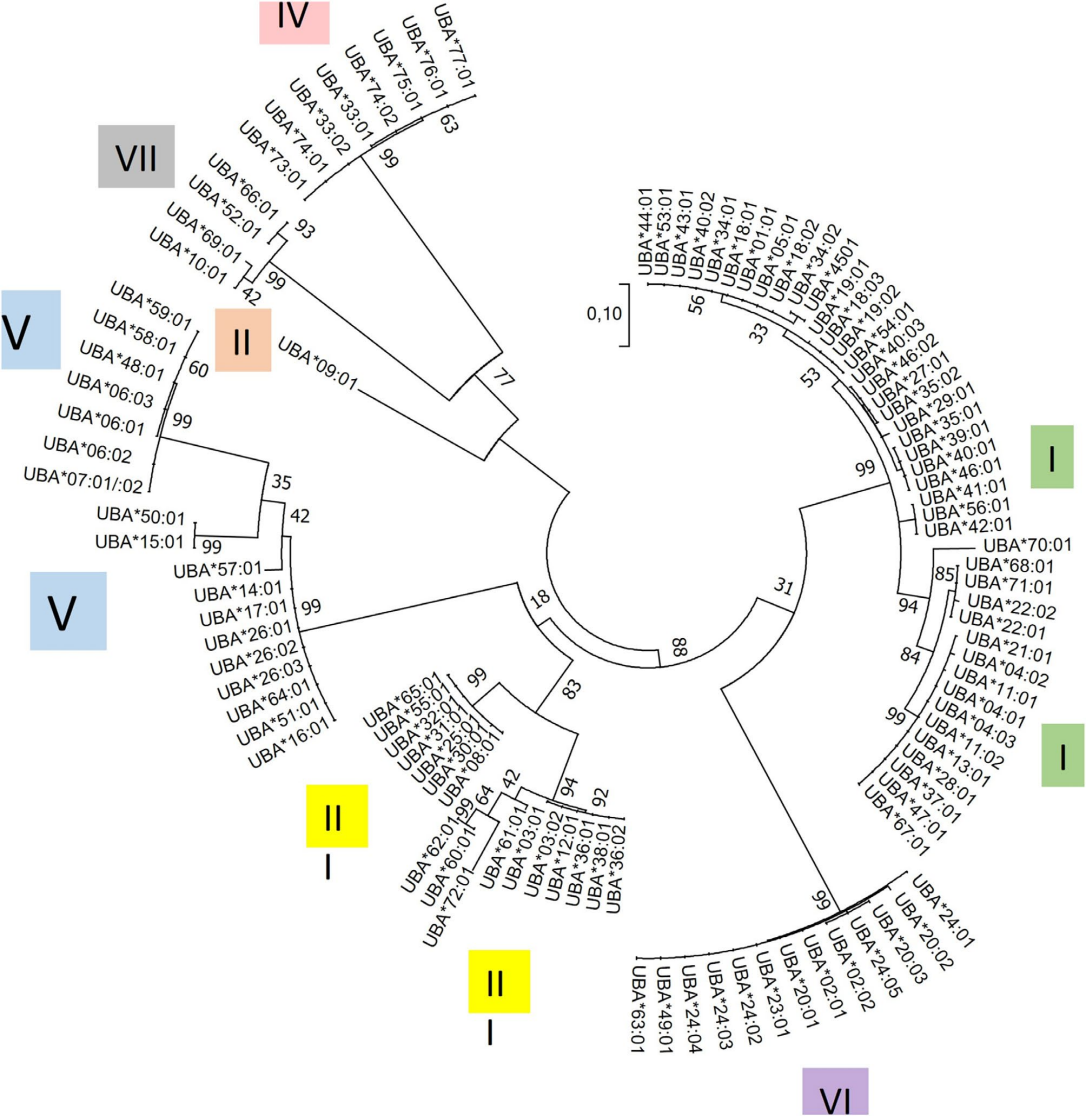
Phylogeny of *UBA* alpha 1 domain sequences. *UBA* alpha 1 domain nucleotide sequences from Hardanger alleles as well as Atlantic salmon alleles present in the IPD‐MHC database are used in the alignment. Seven of the eight defined alpha 1 domain lineages, shared amongst distantly related species (Aoyagi et al. 2002; Grimholt et al. 2015; Kiryu et al. 2005), are shown using roman numbers and different colored boxes. The eight (VIII) alpha 1 domain lineage has so far only been described in rainbow trout and is not included. The tree with the highest log likelihood (−2821,57) is shown. The percentage of trees in which the associated taxa clustered together is shown next to the branches. A discrete Gamma distribution was used to model evolutionary rate differences among sites (5 categories (+*G*, parameter = 23,836)). The tree is drawn to scale, with branch lengths measured in the number of substitutions per site. The analysis involved 104 nucleotide sequences. Codon positions included were 1st + 2nd + 3rd + Noncoding. There was a total of 236 positions in the final dataset.

**FIGURE 3:**
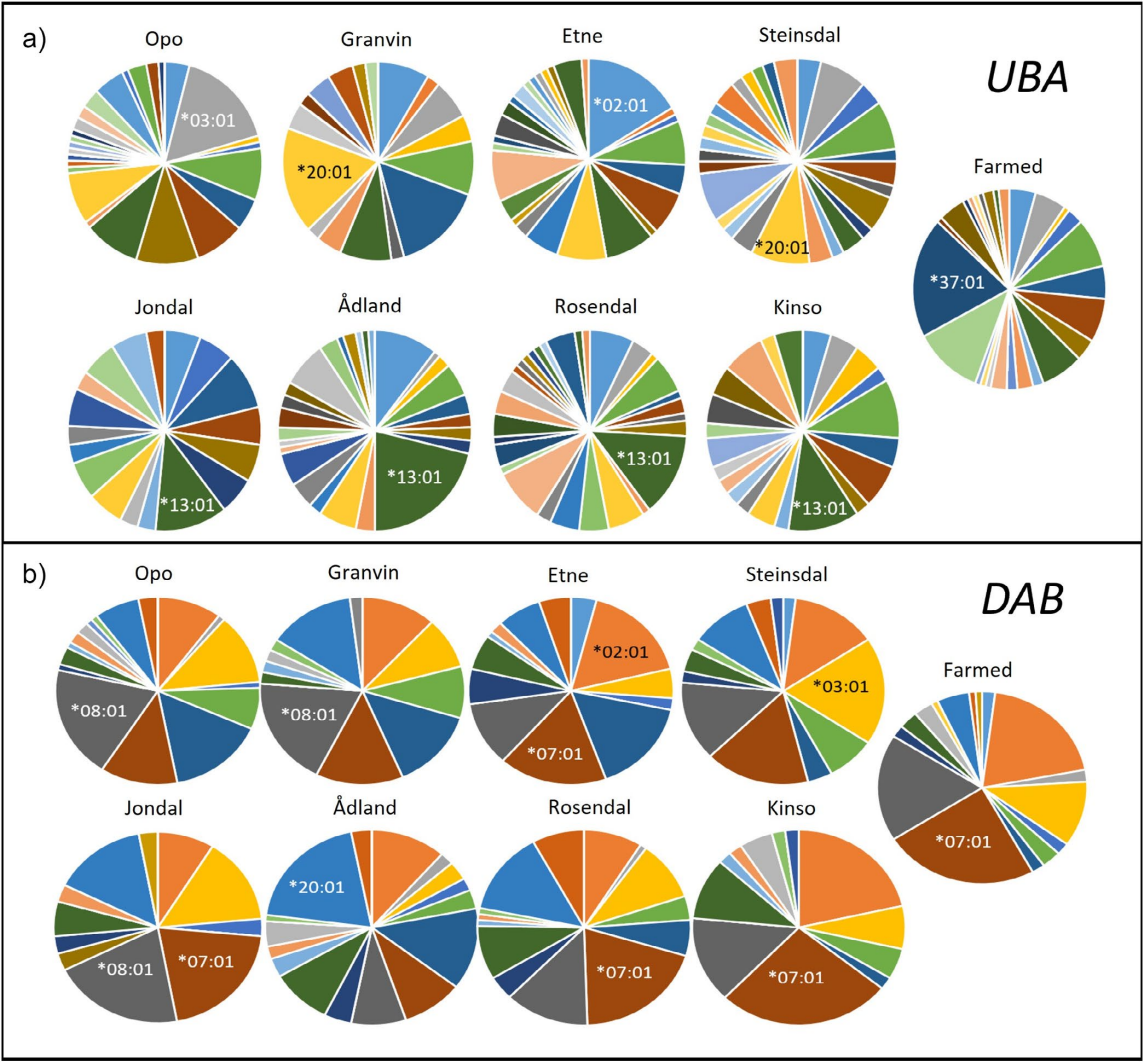
Sector diagrams showing frequencies for (a) *UBA* and (b) *DAB* alleles in the eight wild and one farmed escapee samples. The most frequent allele(s) are shown in each sample. The figure is based on individual allele frequencies shown in Appendix S1.3.

With two individuals lacking genotypes, we found 69 *UBA* alleles in 263 of our 265 wild Atlantic salmon where 53 allele sequences were new, i.e., not recorded in the IPD‐MHC database (Figure 2, Appendix S1.2–1.5). The number of *UBA* alleles per population ranged from 18 in Granvin and Jondal to 31 in Rosendal (Table 2). Allele frequencies for the different wild populations and the farmed group are shown in Figure 3 and detailed in Appendix S1.3. Using rarefied allelic richness estimates (based on a sample size of 17) to compare *UBA* diversity between wild populations showed a diversity ranging from 17.37 in Granvin to 22.46 in Steinsdal (Table 2). We found 30 different alleles among 88 farmed escapees. Three alleles were unique to the farmed group (Appendix S1.3). Rarefied allelic richness (based on a sample size of 17) among all farmed escapees, encompassing multiple years and several independent escape events, was 17.34. In contrast, allelic richness for the escapees from River Etne, representing one single escape event, was 13.89 (Table 2).

**TABLE 2:** Heterozygosity and allelic richness for *DAB* and *UBA* genes.

	Population	*N*_ind_	*N*_all_	H_O_	H_E_	A_R_
*UBA*	Granvin	22	18	0.636	0.907	17.37
	Jondal	17	18	0.647	0.931	18.00
	Kinso	21	22	0.619	0.941	21.66
	Rosendal	40	31	0.800	0.943	19.72
	Ådland	45	27	0.755	0.917	17.90
	Etne	40	30	0.875	0.942	18.68
	Opo	52	29	0.750	0.932	20.59
	Steinsdal	26	29	0.815	0.954	22.46
	Escapees	88	30	0.670	0.937	17.34
	Escapees Etne	33	18	0.727	0.891	13.89
*DAB*	Granvin	22	12	0.954	0.879	11.76
	Jondal	17	11	0.823	0.856	11.00
	Kinso	21	12	0.952	0.843	11.83
	Rosendal	40	14	0.950	0.887	11.20
	Ådland	45	16	0.955	0.901	13.04
	Etne	41	13	0.900	0.887	11.54
	Opo	49	17	0.923	0.895	11.91
	Steinsdal	26	13	0.852	0.883	11.25
	Escapees	88	16	0.784	0.854	11.18
	Escapees Etne	34	11	0.853	0.821	9.46

*Note:* Observed (Ho) and expected (He) heterozygosity as well as rarefied allelic richness (A_R_) data are shown for the *DAB* and *UBA* genes. A few individuals had missing genotype. The sample size used for the rarefied allelic richness estimates is 17.

Abbreviations: *N*_all_, number of alleles; *N*_ind_, number of individuals.

To compare allelic diversity in wild salmon as a single group, farmed escapees from multiple independent escape events and years, and farmed escapees from a single escape event, we pooled all samples of wild salmon (*N* = 263) and estimated the rarefied allelic richness using the sample size of the single event of escapees in River Etne (*N* = 33). The rarefied allelic richness was highest in wild salmon (30.94), followed by farmed escapees from multiple independent escape events (22.25), and lowest in farmed escapees from the single escape event (18.00).

Nucleotide sequence identity between new alleles and alleles previously registered in the IPD‐MHC database ranges from 57% to 99% (IPD‐MHC Database, and Appendix S2) and the majority of this diversity resides within the alpha 1 domain. These alpha 1 domain sequences have been defined into eight lineages that are shared between distantly related species (Aoyagi et al. 2002; Grimholt et al. 2015; Kiryu et al. 2005). Our 53 newly identified wild alleles are distributed along six of these eight lineages (Figure 2). The a1 domain lineage I is the most abundant, adding 22 alpha 1 domain lineage sequences to the 18 Atlantic salmon alleles previously shown to share this lineage (Grimholt et al. 2015). As expected, the three forward PCR primers differentially amplify alleles containing these lineages. Due to the high *UBA* diversity, many alleles are present at low frequencies. This results in 21 of the 72 *UBA* alleles being unique to a single sampled river or individual, such as UBA*22:02, UBA*24:04, UBA*24:05 and UBA*28:01 (Appendix S1.3). Four of these alleles are unique to one individual and exist in a homozygous state (UBA*44:01, UBA*47:01, UBA*48:01, UBA*56:01, UBA*68:01) (Appendix S1.6).

There was a significant deviation (*p* < 0.01) from Hardy–Weinberg equilibrium in all but the Etne population (*p* = 0.12) at the *UBA* locus, with an observed heterozygosity much lower than the expected. Of the 33 alleles present in a homozygous state, 14 were low‐frequency alleles found in single individuals in one population only (Appendix S1.6). The highest frequency allele UBA*13:01 was also found in a homozygous state in seven out of nine populations. Within populations, the frequency of homozygous individuals was high, and ranged from 15% to 36.4%. Homozygosity did not differ between the sexes and seemed randomly distributed amongst individual alpha 1 domain lineages (data not shown and Appendix S1.6).

### 3.2 *DAB* , the Classical MHC Class II Beta Gene

There were no significant deviations from Hardy–Weinberg expectations at the *DAB* locus, with a similar level of observed and expected heterozygosity, except for the farmed escapees collected at a broad geographical and temporal scale that showed a deficit of heterozygotes (Table 2).

Our material displayed far fewer MHC class II alleles compared to Class I alleles, with only 22 *DAB* alleles identified in 261 individuals from the eight wild river populations with four individuals lacking genotype. Only four of these alleles were new, i.e., not registered in the IPD‐MHC database (Appendix S1.2–1.5; Appendices S2 and S3). One allele extends the previously published DAB*09:01 allele (GenBank accession # AM299717) and is here denoted DAB*09:01_L for long.

Nucleotide sequence identity between *DAB* alleles is much higher than what is found for *UBA*, ranging from 84% to 100% (Appendix S2). Sequence polymorphism primarily resides in the peptide‐binding beta 1 domains (Appendix S1.2), where there are no defined lineages.

Opo had the highest number of different *DAB* alleles with 17 alleles, while only 11 alleles were observed in Jondal (Table 2). These were also the populations with the highest and lowest number of individuals represented. Overall, the most frequent allele was DAB*07:01, which dominated in the Kinso and Rosendal populations (Figure 3, Appendix S1.3). Allele frequencies for the different wild populations and the farmed group are shown in Figure 3 and detailed in Appendix S1.

Compared to the 22 *DAB* alleles found among wild salmon, we found 16 *DAB* alleles in 88 farmed escapees (Table 2), where one *DAB* allele was unique to the farmed group (Appendices S1.3 and S3).

When considering all wild salmon as one group (*N* = 261), the rarefied allelic richness estimate (based on a sample size of 34) was 14.63 and 13.35 for the escapees (*N* = 88). Corresponding allelic richness of farmed escapees collected in River Etne was 11, and the remaining escapees (*N* = 54) had an allelic richness of 13.83.

### 3.3 *DAA* , the Classical Class II Alpha Gene

We identified 21 DAA alleles in 251 wild salmon where seven alleles were not registered in the IPD‐MHC database (Appendix S1.2–1.5).

Three of the new alleles differed by less than 3 amino acids from previously identified alleles and were thus given the four‐digit extension DAA*xx:02 or DAA*xx:03 (Ballingall et al. 2018). Our amplified sequences do not discriminate between DAA*01:01 and DAA*01:02 alleles differing in one amino acid in the alpha 2 domain outside our sequenced fragment, so these sequences have been defined as one allele in the subsequent analysis (DAA*01:0x). One allele matched the DAA*03:03:01 sequence (GenBank accession # AY780909) but has for analysis purposes been defined as DAA*03:03 in Appendices [Link], [Link], [Link] to comply with a four‐digit analysis.

The number of alleles per river ranged from 9 in Jondal to 15 in Opo. The most frequent allele was DAA*09:01 dominating in the Etne, Rosendal, and Steinsdal populations with a record high frequency of 35.7% in Kinso (Appendix S1.3). Allele frequencies for the different wild populations and the farmed group are detailed in Appendix S1.

In comparison to the 21 DAA alleles in wild salmon, we found 13 DAA alleles in the escapees, where two DAA alleles were new and unique to this sample collection (Appendix S1.3). Allelic richness for DAA is expected to be identical to that calculated for DAB as these alleles co‐segregate as haplotypes being very closely linked (see below).

### 3.4 *DAA* ‐ *DAB* Haplotypes

Atlantic salmon *DAA* and *DAB* genes are closely linked, and alleles segregate as functional haplotypes (Stet et al. 2002). This is also visible in the current material, where for instance DAB*03:01 consistently segregates with DAA*05:01 in wild as well as in farmed individuals (Appendices S1.3 and S3). Disregarding allele combinations found in single individuals that could be typing artefacts, some haplotypes do not show consistent segregation of specific alleles. For instance, the DAA*03:02 allele segregates with either DAB*20:01 or DAB*20:02. These two haplotypes have most likely arisen due to a single base mutation in the *DAB* gene. Similar examples of single nucleotide substitutions changing the haplotype is DAA*04:01 segregating with either DAB*09:01 or DAB*09:02. Other inconsistent haplotypes suggest that either recombination or gene conversion have influenced allele combinations. The MHCII beta allele DAB*02:01 segregates with either DAA*02:01 or DAA*09:01 in many wild as well as farmed salmon. There are 5 amino acid substitutions between DAA*02:01 and DAA*09:01, not supporting single gene mutations causing the shift. Another *DAB* allele, DAB*09:01, also segregates with two alternative *DAA* alleles, i.e., DAA*04:01 or DAA*09:01 in wild salmon. Also here, there are 5 amino acid differences between the two *DAA* alleles suggesting that other mechanisms than single nucleotide substitutions have provided the haplotype change. If recombination caused the shift, it has occurred within a three‐kilo base region separating the *DAA* and *DAB* genes according to the NCBI genome JAIUJH010000000 and scaffold JAIUJH010001024.1.

Despite several attempts, we lack data on *DAA*‐*DAB* haplotypes in one wild individual, i.e., OPO19 (Appendix S3), where we could not amplify *DAB* alleles segregating with the *DAA* alleles DAA*15:01 and DAA*16:01. This could be caused by sequence diversity in the forward primer region.

### 3.5 Comparing Populations

The genetic differences between populations (*F*_ST_) were found to be very low, although significant at the *DAB* locus (0.7%), the *UBA* locus (0.9%), and for the 68 putatively neutral loci (0.6%) (Appendix S1.7). This implies that natural selection is not acting strongly in different directions across populations at the MHC‐loci. In contrast, the corresponding *F*_ST_‐values for the Vgll3 and the Six6 locus, known to be associated with age at maturity and subjected to strong natural selection (Barson et al. 2015), were substantially higher, at 6.4% and 9.4%, respectively. This pattern suggests a pronounced potential for local adaptation at genes under strong selective pressure in the studied populations.

**FIGURE 4:**
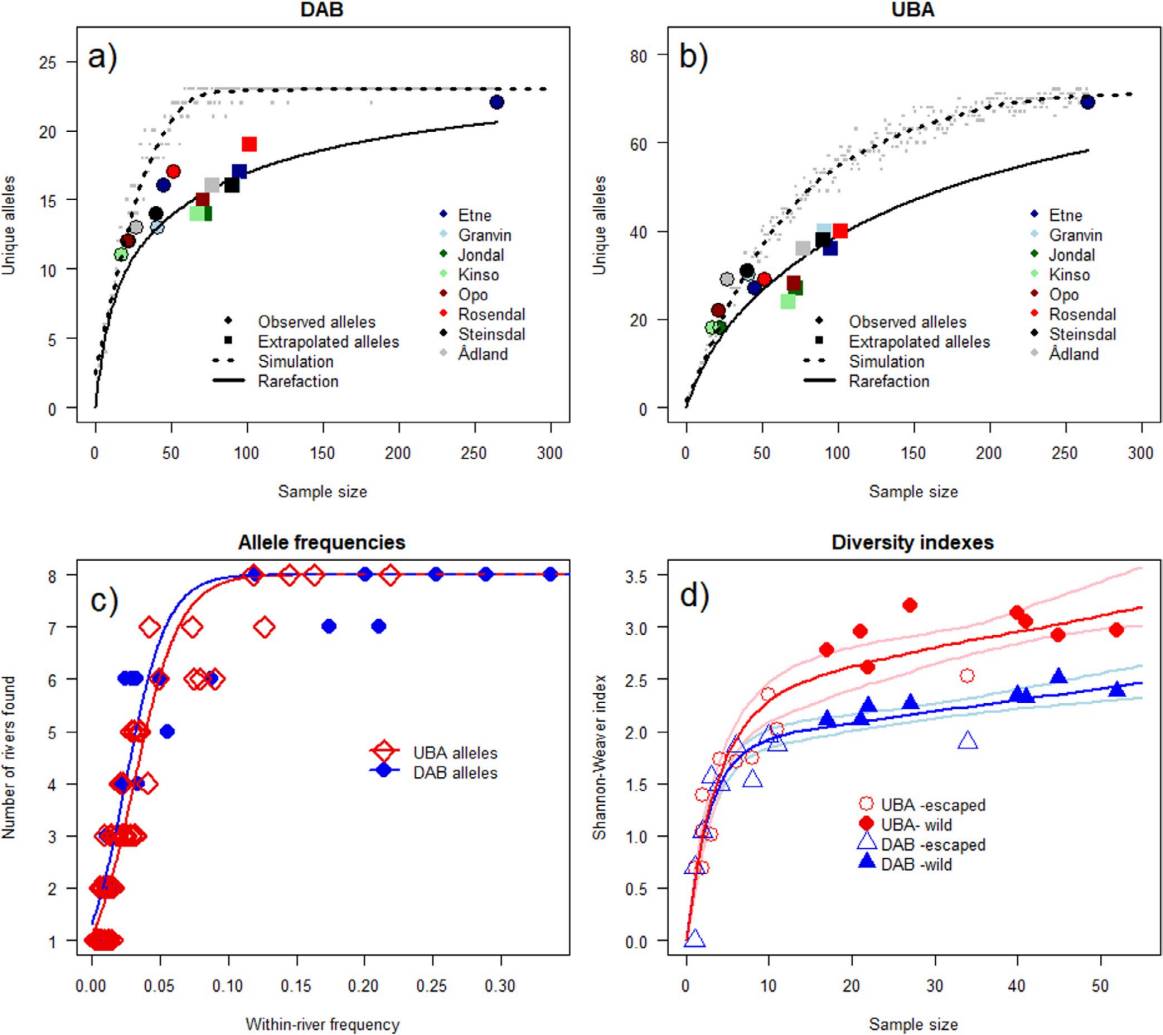
(a, b) Observed number of *DAB* and *UBA* alleles from wild populations in the eight sampled rivers and total population (points) as a function of sample size, together with expected total number of alleles from extrapolation analysis per river (squares). (c) The mean frequency of each allele for the rivers in which it is found versus the number of rivers where the allele has been identified. We see a strong relationship suggesting that alleles that are common in one river population tend to be found in all rivers, but rare alleles are likely to be unique to one or a few rivers. (d) Diversity indexes for all samples, both wild (solid symbols) and farm‐originated (open symbols) as a function of sample sizes. Diversity increases sharply with sample size, declining towards an asymptote as a more complete sample is achieved. Most samples of farm stock are so small that their diversity is strongly correlated with sample sizes as new alleles are rapidly accumulated with each new individual. Only the Etne river (rightmost blue/red open symbols) is large enough that accumulation rates have tapered off for wild samples. The diversity indices for the Etne farm‐derived samples are markedly lower than the range of comparable wild samples, but with only one sample of sufficient size more studies are needed.

Rarefaction analyses combined with stochastic simulation may suggest that most *UBA* and *DAB* alleles in the wild population are represented in the sampled individuals, as the accumulation curves for simulating samples that best match the observed curves form asymptotes for the rarefaction analyses (Figure 4a,b).

However, the alleles that are rare within each population also tend to be found in only one or two out of the eight populations. Rarefaction analysis shows that an extra 2 (1–3) *DAB* alleles and 8 (7–11) *UBA* alleles are likely to exist per population at low frequencies. When extrapolating this expectation to all eight populations, an additional 8 rare *DAB* alleles and 30 rare *UBA* alleles would be expected in total (Figure 4c). We see a strong relationship suggesting that alleles common in one population tend to be found in all populations, but rare alleles are likely to be unique to one or a few populations. Diversity indexes for all samples, both wild and farm‐originated river samples, increase sharply with sample size, especially for small samples, approaching an asymptote as a more complete sample is achieved. Most samples of farm stock are so small that diversity is strongly correlated with sample sizes as new alleles are rapidly accumulated with each new individual, only the Etne river being large enough that accumulation rates have tapered off for wild samples (see Figure 4). The diversity indices for the Etne farm‐derived samples are markedly lower than the range of comparable wild samples, but with only one sample of sufficient size, more studies are needed to substantiate and quantify differences in genetic diversity and hence the effects of interbreeding.

The Shannon‐Weaver index is not saturated but tightly correlated with sample size (*r* = 0.82, *p* < 0.0001). There is no significant difference in allele diversity index between sexes. Comparing the diversities of escaped farmed salmon and wild populations is difficult in this material, as the sample size per site is low, especially for the farmed salmon. Thus, while the average diversity index is much lower for farmed salmon per river than for wild salmon, the accumulation curves seem consistent with all farmed and wild populations following the same diversity curve as a function of sample size (Figure 4d). The only sample of farmed fish with a comparable sample size is from the river Etne (*n* = 34 farmed, *n* = 41 wild), and the diversity for both *DAB* and *UBA* alleles there is lower than expected for the sample size from leave‐half‐out bootstrapping a confidence interval for the Michaelis–Menten accumulation curves. However, while the Etne river sample fits on a saturation curve whose lower asymptotic diversity suggests a notably lower MHC diversity in the farmed escapees than wild salmon, more samples of size 30+ from more rivers would be needed to extrapolate with certainty.

## 4 Discussion

The eight wild populations in Hardanger displayed 9–15 *DAA* alleles, 11–17 *DAB* alleles and 18–31 *UBA* alleles (Table 2, Appendix S1.3). Farmed individuals displayed a similar diversity with 13 *DAA*, 16 *DAB* and 30 *UBA* alleles. Adjusting for sample size, allelic richness (A_R_) was at similar level in the wild populations, except for Ådland that had a higher allelic richness in the *DAB* locus, but a low level of allelic richness in the UBA locus. Collectively, the allelic richness in the farmed escapees were at the same level as the in wild populations, but considerably lower in the escapees from a single event of escapes caught in River Etne.

In our study, the samples of farmed salmon were collected as escaped farmed salmon from a wide range of rivers over several years. We therefore expect that these fish represent unique strains from many breeding companies. Genetic assignment of the farmed escapees to the original escape events or breeding lines could however not be conducted due to a lack of genetic references.

The farm breeding strains in Norway were sourced from many wild populations, mainly from Mid‐ and Western‐Norway (Gjedrem et al. 1991; Gjøen and Bentsen 1997), but there are no records of the breeding strains being sourced from the eight populations analysed in the present study. Because of the limited number of wild founders and consecutive genetic drift, the breeding strains have lost genetic variation (Karlsson et al. 2010; Skaala et al. 2004). Single farms contain fish from specific breeding strains and often with fish from a very restricted number of parents. Escaped farmed salmon from a single farm may therefore have a considerably lower genetic diversity than a representative sample of all farmed salmon. This was supported in our study, where the escapees in river Etne had considerably lower allelic richness compared to the escapees sampled in many different locations and years. However, compared to all wild salmon analysed in the present study, also the broadly sampled escaped farmed salmon had considerably lower allelic richness. Consequently, if farmed salmon from a single escape event causes substantial genetic introgression, this may significantly affect MHC diversity in wild populations. Repeated introgression from numerous farmed strains is also expected to progressively reduce and homogenize MHC diversity in wild populations.

In addition, selection of MHC genes has been documented between North American breeding lines and one of the breeding lines in Norway (Mowi) (Buso et al. 2025) where farmed salmon have gone through recent selective sweeps while wild salmon show signs of long‐term balancing selection. This indicates that the MHC diversity has changed from its wild origin not only from founder effects and genetic drift but also from a different selection regime compared to selection in nature. Future analyses of the MHC diversity in many of the breeding lines in use in Norway, and in wild salmon admixed with escaped farmed salmon, will enable us to better understand and quantify the impact farmed genetic introgression has on immune diversity and the ability to combat future pathogens.

Using our three different forward primers, we amplified 72 different *UBA* alleles in the wild and farmed groups, covering what we believe is the entire diversity of this gene in these individuals. These three forward primers amplified alleles from all alpha 1 domain lineages shown to be present in Atlantic salmon (Grimholt et al. 2015). The eight (VIII) alpha 1 domain lineage has so far only been described in rainbow trout (Kiryu et al. 2005). For both MHC class II genes, single forward primers are sufficient to amplify all 22 *DAB* and 21 *DAA* alleles, as shown previously (Stet et al. 2002; Sundaram et al. 2020).

We identified approximately three times the number of *UBA* alleles compared to *DAA* and *DAB* alleles in the wild Atlantic salmon, i.e., 69 *UBA*, 21 *DAA* and 22 *DAB* alleles in the eight populations. Our observation is in line with previous studies of Atlantic salmon populations, showing that the MHC class I is more polymorphic than MHC class II. A pilot study of nine individuals from another endangered Norwegian population (Vosso), found twelve *UBA* alleles and eight *DAB* alleles (Sundaram et al. 2020). A study of MHC diversity in Irish and Norwegian wild Atlantic salmon displayed 21 *UBA* and 14 *DAA* alleles in 19 Irish individuals from four rivers and 13 *UBA* and 14 *DAA* alleles in 34 Norwegian individuals from four rivers, of which none are located in the Hardanger region (Consuegra, Megens, Leon, et al. 2005; Consuegra, Megens, Schaschl, et al. 2005). Nevertheless, higher MHCI than MHCII diversity may be a general trend in this species.

Threats from aquaculture in the form of sea lice and escapees (genetic introgression) were the background for the establishment of a dedicated gene bank for wild salmon in the Hardanger region. In this study, the MHC diversity in Atlantic salmon used as founders in the gene bank program was studied. Accordingly, only salmon that were defined as being of wild origin were included. Although there are large uncertainties at the individual level in identifying farmed ancestry (Karlsson et al. 2014), the genetic influence of farm escapees on MHC diversity has been reduced as much as possible with currently available methods. Wild salmon in the Hardanger region have a very high degree of genetic introgression from farmed salmon (Diserud et al. 2022, 2023; Karlsson et al. 2016). This is illustrated by the proportion of captured salmon that are discarded as broodstock for the gene bank program due to genetic introgression from farmed salmon (Karlsson et al. 2025). Despite the efforts to exclude broodfish with farmed ancestors, the introduction of MHC alleles through genetic introgression from farmed salmon cannot be ruled out.

The study was initiated due to concerns about immune protection in the Hardanger salmon populations, many of which are in poor state and threatened by negative environmental and anthropogenic factors (Forseth et al. 2017). In comparison with the limited number of studies that have investigated MHC diversity in wild Atlantic salmon (Consuegra, Megens, Leon, et al. 2005; Consuegra, Megens, Schaschl, et al. 2005; Sundaram et al. 2020), the Hardanger populations display a medium to high MHC diversity. Factors such as straying, susceptibility to farmed genetic introgression, pathogen‐ driven balancing selection and MHC‐influenced mate choice are possible sources shaping the diversity. Future analyses of MHC‐diversity, also including populations in good state, is warranted to disentangle the effect different anthropogenetic changes and natural variation of habitats may have on MHC diversity. The rate of straying, i.e., when salmon return to non‐natal rivers to spawn, varies with time and space, but is also dependent on factors pertaining to natural vs. hatchery reared origin of the salmon and the hydrological and geographical characteristics of the rivers in question (Jonsson et al. 2003; Stabell 1984). Straying occurs at a lower rate (3%–6%) in naturally recruited populations than after released hatchery‐reared smolt (15%) (Jonsson et al. 2003) and rivers with higher water flow are more attractive to straying salmon than smaller rivers with low flow rate. Small populations are also more easily impacted by introgression from either straying wild salmon or escaped farmed salmon, and this effect is especially prominent when the natal population is reduced due to environmental impacts. Straying may be a major factor causing the low genetic differentiation we observed between the wild populations in this fjord. Interestingly we observed the same level of genetic differentiation at the MHC‐loci as with the 68 neutral SNPs. This implies that the structure of genetic variation at MHC between populations is not strongly driven by different directions of natural selection in the different rivers. In contrast the Vgll3 and the Six6 markers that is tightly linked to age at maturity showed very large differences between populations, implying a sufficient genetic isolation for local adaptation at genes having strong functional effects.

In addition to straying, pathogen‐driven balancing selection and MHC‐influenced mate choice are possible additional sources that may increase diversity. Mating choice as a driving force for increased MHC diversity has been investigated in multiple salmonids with various results (reviewed in Auld et al. 2019). Some studies have concluded that Atlantic salmon choose mates with dissimilar MHC to themselves (Consuegra and de Leaniz 2008; Evans et al. 2012; Landry et al. 2001). Other studies found no effect, greater male fertilization success when heterozygous at MHC alleles or even higher preference for similar MHC mates (Skarstein et al. 2005; Weir et al. 2012; Yeates et al. 2009). If these diversities are caused by differences in methodology, or a biological difference between species and/or populations, remains to be established.

MHC has been implicated in providing resistance against pathogens in salmonids. A well‐supported link between *DAB* and resistance towards the bacterium *Aeromonas salmonicida* has been shown for Atlantic salmon as well as Arctic charr ( *Salvelinus alpinus* ) (Croisetiere et al. 2008; Grimholt et al. 2003; Kjoglum et al. 2008; Langefors et al. 2001; Lohm et al. 2002). A few studies have shown an effect of the classical class I locus on resistance to the pathogens Infectious Anaemia Virus (ISAV) (Grimholt et al. 2003; Kjoglum et al. 2006) and Piscine Myocarditis Virus (PMCV) (Hillestad and Moghadam 2019; Hillestad et al. 2020; Mogahadam and Rosaeg 2023). For PMCV, one of the two major QTLs resides within a 500 kilobase region on chromosome 27, with the major classical MHC class I locus *UBA* as the only polymorphic causative gene within this region (Grimholt 2018). PMCV‐positive individuals were detected in the three rivers Rosendal, Ådland and Opo, so it is likely that other Hardanger salmon populations have also been exposed to this pathogen. However, how extensive the pressure from PMCV and other pathogens has been in this region is unknown.

Possible pathogen‐driven changes in existing alleles can be seen on a molecular level. Single point mutations occur in both *UBA* as well as *DAB* and *DAA* as exemplified by some of the *0x:02 and *0x:03 alleles. An additional mechanism for generating new alleles is operational for *UBA* where alpha 1 domains are shuffled between alpha 2 and downstream domains producing new alleles (Grimholt et al. 2015). This phenomenon is also seen for many of the other UBA alleles that share 100% identity in the alpha 1 domain, e.g., UBA*08:01, UBA*25:01, UBA*30:01, UBA*31:01, UBA*32:01, UBA*55:01 and UBA*65:01, all of which belong to the alpha 1 domain lineage III (Figure 1). At least some of these recombinations are widespread and potentially old, such as the *UBA* alleles *06:01 and *07:01 found in the Hardanger region, but also in a farmed population, as well as in animals from the Vosso river (Grimholt et al. 2003; Sundaram et al. 2020).

Pathogen driven selection can increase MHC diversity where the three main mechanisms proposed are heterozygote advantage, fluctuating selection, and negative frequency‐dependent selection (reviewed in Radwan et al. 2020). None of these three mechanisms has currently been sufficient to explain all aspects of MHC diversity, although the current view is in favour of heterozygote advantage and negative frequency‐dependent selection mechanisms.

Heterozygous advantage, particularly in a species with a single classical MHC gene, would double the chance of presenting immunogenic peptides. *DAB* showed no difference between expected versus observed heterozygosity, but this was not the case for *UBA* (Table 2). Despite confidence that we amplified all alleles present, the *UBA* gene displayed significant deviation from Hardy–Weinberg equilibrium due to excess homozygosity in all but the Etne population (Table 2). Each presented *UBA* allele was supported by many Illumina reads, and polymorphic residues comply with other patterns, suggesting they are not artefacts. If homozygosity is favorable in infection of specific pathogens, one would expect a few alleles to dominate, however the alleles seem randomly distributed also amongst individual alpha 1 domain lineages.

The heterozygote advantage hypothesis has been expanded with the view of MHCI alleles as being specialists or generalists in humans as well as in chickens [reviewed in (Kaufman 2018)]. Some MHCI alleles are only capable of presenting one or a few peptides, being so‐called specialists, while others have a broad peptide binding potential and are denoted generalists. For Marek's disease in chickens, a generalist MHCI allele was linked to resistance, while non‐progression from HIV to AIDS in humans was linked to a specialist MHCI allele. So far, we have no data on the peptide‐binding ability of salmonid MHCI alleles, but it would not be surprising if salmonid MHCI alleles also comply with this specialist and generalist view. As opposed to higher vertebrates, salmonid single classical MHC class I (*UBA*) and single classical MHC class II (*DAA*‐*DAB*) genes reside on different chromosomes (Lukacs et al. 2010), enabling selective forces to act independently on the two classes of genes.

Viruses have developed numerous strategies to avoid MHC presentation and triggering of protective T cell responses (Hansen and Bouvier 2009; Schuren et al. 2016), which provides the foundation for the negative frequency‐dependent selection mechanism. Changes in pathogens need to be met by changes in the host to enable continued immune protection. Pathogens adapt to or evade existing MHC alleles, thus driving changes in existing alleles. Shuffling of alpha 1 domains to create new allelic MHCI sequences is one such approach, which is not unique to salmonids. For instance, zebrafish and salmonids, which separated 250 million years ago, share multiple highly divergent alpha 1 domain sequences representing ancient domain lineages that are shuffled onto variable alpha 2 plus downstream sequences (Grimholt et al. 2015; Kiryu et al. 2005; Nonaka et al. 2011). The role of pathogen selection on such transspecies polymorphism is more controversial (Radwan et al. 2020), but Bolnick and Stutz (2017) showed how this mechanism provides a functional advantage for rare MHC alleles in stickleback.

Our study implies that homozygous advantage for MHCI is not unique to wild individuals but also found in farmed salmon. We found 32% of the farmed escapees to be homozygous for *UBA*. These individuals have been heavily selected for breeding traits such as growth, meat quality, and feed uptake and for a general robustness against the most common pathogens. However, these escapees represent farmed salmon from a broad geographical range (Figure 1, Appendix S1.02), most likely originating from several, if not all, major Norwegian breeding strains (Aqua Gen, MOWI, Salmo Breed, and Rauma), which are genetically isolated; hence, the observed excess of homozygotes can therefore also be explained by a Wahlund effect (Wahlund 1928). Future analyses of the separate breeding strains will shed more light on the mechanisms behind maintaining MHC diversity in farmed salmon.

Increased homozygosity could be a functional benefit from the MHC alleles themselves or result from co‐segregation with other closely linked genes. Contrary to chickens, where the classical MHCI gene is closely linked to polymorphic Transport Associated Protein 2 (*TAP2*) and TAP Binding Protein (*TAPBP*) genes, Atlantic salmon does not display such polymorphism in closely linked genes of relevance for antigen presentation (Grimholt 2018). Polymorphism in genes outside the core MHCI regions has so far not been studied, so these regions could contain candidate genes promoting *UBA* homozygosity. However, the number of homozygous individuals relates to allele frequencies and not to specific alleles or alleles with specific alpha 1 domain lineages, suggesting other yet unknown mechanisms are operational.

High MHC‐diversity is important for the long‐term adaptation of wild salmon populations and therefore highly relevant for conservation programs. Over the years, the target number of founder fish (wild broodstock) in the gene bank program has increased from 50 to 100 individuals per population, aiming to maintain adequate genetic diversity. Our findings support the need to further increase this number to 200 founders to capture rare MHC alleles. Moreover, many of these rare alleles were unique to one or two rivers, showing the importance of including multiple rivers within a region in conservation programs not just increasing the number of founders from large populations.

## 5 Conclusion

This first large‐scale investigation of allelic MHC diversity in wild Atlantic salmon populations in Norway reveals substantial MHC variation within the sampled Hardanger region populations. The salmon populations in the Hardanger region are highly impacted by aquaculture and genetic introgression of escaped farmed salmon is a major threat as it compromises the genetic variation and genetic integrity of the wild populations. We demonstrated that escaped farmed salmon have lower MHC diversity than the wild salmon in the region. Accumulation of farmed to wild genetic introgression is expected to reduce MHC diversity within and among populations.

Because MHC diversity is directly linked to disease resistance, populations with diverse MHC alleles are more likely to adapt to changing environments with emerging pathogens. Preservation of high MHC diversity within and among populations in the Atlantic salmon gene bank program is thus an important goal. To achieve this, a large number of founders is important, and MHC genotyping may also aid in the inclusion of rare or underrepresented alleles.

## Funding

This study was funded by the strategic institute project 10421 at the Norwegian Veterinary Institute, and the Norwegian Research Council project no. 274635. ÅHG and HL were supported by the Norwegian Environment Agency.

## Ethics Statement

None of the Atlantic salmon were euthanized specifically for this study. Samples from wild and escaped farmed salmon captured in rivers represent secondary use of biobank material collected through the mandatory health control of wild anadromous salmon in the national gene bank program and stock enhancement hatcheries. These were anesthetized using benzocaine or metacaine and euthanized by exsanguination. Samples from escaped farmed salmon captured at sea were obtained during licensed coastal fisheries, where fish were euthanized by a forceful blow to the head followed by bleeding from the gill arches.

## Conflicts of Interest

The authors declare no conflicts of interest.

## Supporting information


**Appendix S1:** Additional data: Details on sampling, primers and MHC sequences.

**Appendix S2:** Percent identity MHC: Sequence identity between MHC alleles. Nucleotide sequence identity for UBA alpha1, DAB, and DAA and amino acid sequence identity for UBA alpha 1 between sequences from this study in addition to sequences registered in the IPD‐MHC database (IPD‐MHC Database) calculated using ClustalX2 (Larkin et al. 2007).

**Appendix S3:** MHC alleles each animal: UBA, DAB, and DAA alleles in addition to sex for each of the wild and farmed escapee animals.

## Data Availability

The data that support the findings of this study are openly available in NCBI SRA at https://www.ncbi.nlm.nih.gov/sra, reference number PRJNA578031 and PRJNA1097785.
