# Supplementary material for: Farmed Escapees Threaten MHC Diversity in Wild Atlantic Salmon
Source: Evol Appl. 2026 May 29;19(6):e70278. doi: 10.1111/eva.70278 (PMC13238831; doi:10.1111/eva.70278)
Supplement: Supplementary file 1 — Appendix S1: Additional data: Details on sampling, primers and MHC sequences. [file EVA-19-e70278-s001.docx]

Supplementary File 1 (SF1). Additional data

| Table of Content | | Page |
| --- | --- | --- |
| SF1.01 | Quality norm and impact factor | 2 |
| SF1.02 | Sampling data for farmed escapees | 3 |
| SF1.1 | Amplicon primer sequences | 4 |
| SF1.2 | Alignments of deduced MHC amino acid sequences | 4 |
|  | Alignment of *UBA* sequences | 5 |
|  | Alignment of *DAB* sequences | 10 |
|  | Alignment of *DAA* sequences | 13 |
| SF1.3 | MHC allele distribution and *DAA*-*DAB* haplotypes | 14 |
|  | *UBA* allele frequencies | 14 |
|  | *DAB* allele frequencies | 16 |
|  | *DAA* allele frequencies | 17 |
|  | *DAA*-*DAB* haplotypes | 18 |
| SF1.4 | Nucleotide sequence phylogenies | 19 |
|  | Phylogeny of *UBA* sequences | 20 |
|  | Phylogeny of *DAB* sequences | 21 |
|  | Phylogeny of *DAA* sequences | 22 |
| SF1.5 | Accession numbers for new alleles | 23 |
| SF1.6 | UBA homozygosity | 24 |
| SF1.7 | Fst values | 25 |

Table SF1.01. Quality norm and impact factor assessment for the period 2015-2019

|  | 041.Z Etne | 044.3Z Ådland | 045.4Z  Rosendal | 047.2Z Jondal | 048.Z  Opo including Lake Sandvin | 050.1Z  Kinso | 052.1Z  Granvin | 052.7Z  Steinsdal |
| --- | --- | --- | --- | --- | --- | --- | --- | --- |
| Spawning target (kg females) | 1025 | Not determined | 99 | 54 | 798 | 126 | 187 | 233 |
| Classification (quality norm =1, simplified classification=2) | 1 | 2 | 1 | 2 | 2 | 1 | 1 | 1 |
| Attainment of spawning target % (2015-2019) | 100 |  | 90 |  |  | 84 | 99 | 53 |
| Harvestable surplus (% of normal) | 105 |  | 40 |  |  | 38 | 42 | 0 |
| State based on attainment of spawning target and harvestable surplus | VG | P/VP | VP | P/VP | P/VP | VP | P | VP |
| State: Genetic integrity | VP | VP | VP | VP | VP | VP | VP | VP |
| Overall assessment (according to quality norm or the simplified assessment) | VP | VP | VP | VP | P/VP | VP | VP | VP |
| Transportation/infrastructure impact | 0 | 0 | 0 | 1 | 0 | 0 | 0 | 1 |
| Habitat alteration (impact) | 0 | 0 | 1 | 1 | 2 | 0 | 0 | 0 |
| Agricultural pollution (impact) | 0 | 0 | 0 | 0 | 0 | 0 | 0 | 1 |
| Developed for hydropower (year) | 1963 | 1934 | 1957 | 1974 |  |  | 2008 |  |
| Hydropower regulation (impact) | 2 | 0 | 1 | 2 | 0 | 0 | 0 | 0 |
| Other use of riverwater (1=yes, 0 = no) | 0 | 1 | 0 | 0 | 0 | 0 | 0 | 0 |
| Water abstraction (impact) | 0 | 1 | 0 | 0 | 0 | 0 | 0 | 0 |
| Salmon lice (impact) | 2 | 2 | 3 | 3 | 3 | 3 | 3 | 3 |
| Escaped farmed salmon (impact) | 1 | 2 | 2 | 2 | 2 | 2 | 2 | 2 |
| Pink salmon (impact) | 1 | 0 | 0 | 0 | 0 | 0 | 1 | 1 |
| Overexploitation (impact) | 0 |  | 1 |  |  | 1 | 0 | 1 |

Abbreviations are as follows: VG= Very good, P= poor, VP= very poor, VL= very low. The table displays the state of the included Atlantic salmon populations classified according to the quality norm (five populations) or a simplified classification system (four populations) (main text references: Norwegian Ministry of Climate and Environment, 2013; Scientific Advisory Committee for Atlantic Salmon Management, 2021). Rivers are identified with their unique code [e.g. 041.Z for ETNE, The Norwegian Water Resources and Energy Directorate (NVE)] and name. The assessment is based on the achievement of spawning targets, harvest potential, genetic integrity, as well as an overall assessment of the state of the stock. Several anthropogenic activities (i.e. Agricultural pollution, Hydropower regulation, Water abstraction etc) have been assessed and scored as having no effect (0), small effect (1), moderate effect (2) or large effect (3) on population size. The impact factors hazardous substances, acidification and wastewater were scored as having no effect (0) on included populations and are omitted from the table. The classification is based on data from the period 2015 to 2019, and in the overall assessment, all populations were classified as being in a poor or very poor state. In addition, all stocks were impacted by salmon lice and escaped farmed salmon (Adopted from Table 11.5 in main text reference Scientific Advisory Committee for Atlantic Salmon Management, 2021).

Table SF1.02. Sampling data for farmed escapees

| **Population** | **River (R)/Fjord (F)** | **# Samples** | **Year** | North | East |
| --- | --- | --- | --- | --- | --- |
| Drevja | R | 1 | 2009 | 7315208 | 415298 |
| Etne | R | 34 | 2010 | 6649914 | -9586 |
| Fusta | R | 1 | 2009 | 7310339 | 416484 |
| Gaula | R | 1 | 2008 | 7032874 | 261073 |
| Halsan | R | 8 | 2008-2009 | 7304543 | 394692 |
| Lærdal | R | 1 | 2008 | 6797889 | 94547 |
| Moelva | R | 2 | 2008 | 7178746 | 330119 |
| Namsen | R | 4 | 2008 | 7152770 | 331979 |
| Nidelva | R | 6 | 2009 | 7042018 | 271063 |
| Skjomen | R | 1 | 2009 | 7572605 | 598856 |
| Steinkjer | R | 2 | 2008 | 7103090 | 328080 |
| Stjørdal | R | 1 | 2008 | 7042188 | 295871 |
| Surna | R | 3 | 2007+2009 | 6997965 | 178887 |
| Valldal | R | 2 | 2008 | 6931227 | 99212 |
| Vikja | R | 10 | 2008-2009 | 6801973 | 47068 |
| Øyenså | R | 1 | 2008 | 7130707 | 313497 |
| Årøy | R | 2 | 2007 | 6817869 | 80635 |
| Malangen | F | 2 | 2020 | 7705675 | 632056 |
| Rødøy | F | 3 | 2019-2020 | 7382515 | 416803 |
| Høgsfjorden | F | 1 | 2020 | 6561091 | -13577 |
| Namsenfjorden | F | 2 | 2020 | 7166937 | 314173 |
| Andfjorden | F | 1 | 2020 | 7666529 | 538723 |
| Baustaneset | F | 1 | 2020 | 6612716 | -20912 |
| Hasselvika | F | 1 | 2020 | 7067968 | 244129 |
| Total |  | 90 |  |  |  |

Number of samples per river (R) or Fjord (F) site with map coordinates (North and East) are given alongside sampling period.

**SF1.1. Amplicon primer sequences used in this study**

| Primer | Sequence |
| --- | --- |
| First gene specific Illumina PCR primer sets | |
| DAA.F1 | ACACTCTTTCCCTACACGACGCTCTTCCGATCT_TGCTGGCAGGTGTATGCAGAA |
| DAB.F1 | ACACTCTTTCCCTACACGACGCTCTTCCGATCT_ATGTCGATGTCTATCTTCTG |
| UBA.F1 | ACACTCTTTCCCTACACGACGCTCTTCCGATCT_CTGGGAATAGGCCTTCTACAT |
| UBA.F2 | ACACTCTTTCCCTACACGACGCTCTTCCGATCT_AGCCCTACATTCTTCATCTGC |
| UBA.F3 | ACACTCTTTCCCTACACGACGCTCTTCCGATCT_TGCAGTAACCCACTCTCTGA |
| DAA.R | GTGACTGGAGTTCAGACGTGTGCTCTTCCGATCT_GGTGAAATCAGCGTTGGGGT |
| DAB.R | GTGACTGGAGTTCAGACGTGTGCTCTTCCGATCT_GTACCAGTCCCCGTTAGCCAG |
| UBA.R | GTGACTGGAGTTCAGACGTGTGCTCTTCCGATCT_TCCAGATACTTCTTCAGCCA |
|  |  |
| Second PCR primer sets to introduce dual Illumina indexes | |
| PCR2-D-F1 | AATGATACGGCGACCACCGAGATCTACACTTGACTACACTCTTTCCCTACACGAC |
| PCR2-D-F2 | AATGATACGGCGACCACCGAGATCTACACGGAACTACACTCTTTCCCTACACGAC |
| PCR2-D-F3 | AATGATACGGCGACCACCGAGATCTACACTGACATACACTCTTTCCCTACACGAC |
| PCR2-D-F4 | AATGATACGGCGACCACCGAGATCTACACGGACGGACACTCTTTCCCTACACGAC |
| PCR2-D-F5 | AATGATACGGCGACCACCGAGATCTACACCTCTACACACTCTTTCCCTACACGAC |
| PCR2-D-F6 | AATGATACGGCGACCACCGAGATCTACACGCGGACACACTCTTTCCCTACACGAC |
| PCR2-D-F7 | AATGATACGGCGACCACCGAGATCTACACTTTCACACACTCTTTCCCTACACGAC |
| PCR2-D-F8 | AATGATACGGCGACCACCGAGATCTACACGGCCACACACTCTTTCCCTACACGAC |
| PCR2-D-R1 | CAAGCAGAAGACGGCATACGAGATCGTGATGTGACTGGAGTTCAGACGTG |
| PCR2-D-R2 | CAAGCAGAAGACGGCATACGAGATACATCGGTGACTGGAGTTCAGACGTG |
| PCR2-D-R3 | CAAGCAGAAGACGGCATACGAGATGCCTAAGTGACTGGAGTTCAGACGTG |
| PCR2-D-R4 | CAAGCAGAAGACGGCATACGAGATTGGTCAGTGACTGGAGTTCAGACGTG |
| PCR2-D-R5 | CAAGCAGAAGACGGCATACGAGATCACTGTGTGACTGGAGTTCAGACGTG |
| PCR2-D-R6 | CAAGCAGAAGACGGCATACGAGATATTGGCGTGACTGGAGTTCAGACGTG |
| PCR2-D-R7 | CAAGCAGAAGACGGCATACGAGATGATCTGGTGACTGGAGTTCAGACGTG |
| PCR2-D-R8 | CAAGCAGAAGACGGCATACGAGATTCAAGTGTGACTGGAGTTCAGACGTG |
| PCR2-D-R9 | CAAGCAGAAGACGGCATACGAGATCTGATCGTGACTGGAGTTCAGACGTG |
| PCR2-D-R10 | CAAGCAGAAGACGGCATACGAGATAAGCTAGTGACTGGAGTTCAGACGTG |
| PCR2-D-R11 | CAAGCAGAAGACGGCATACGAGATGTAGCCGTGACTGGAGTTCAGACGTG |
| PCR2-D-R12 | CAAGCAGAAGACGGCATACGAGATTACAAGGTGACTGGAGTTCAGACGTG |

**SF1.2. Alignment of deduced MHC amino acid sequences**

In the following sections we compare the new alleles identified in this study with those included in the IPD-MHC database. Accession numbers for new *UBA*, *DAB* and *DAA* alleles are shown in Supplementary file 1.5 while accession numbers for previously identified alleles used in the alignments can be found at the IPD-MHC website (<https://www.ebi.ac.uk/ipd/mhc/group/FISH/>). For all alignments, dashes indicate missing data while dots indicates identity. Abbreviations used are: CP= connecting peptide, TM= transmembrane domain, CYT= cytoplasmic domain. Lines missing data are not shown. Primer regions are shown using green shading. Numbering above the alignment is according to the top sequence. Individual domains are shown above the alignment. Residues are colour coded according to physiochemical properties. New alleles are shown using names with red font.

**Alignment of deduced *UBA* amino acid sequences:**

Alpha 1 domain
 * 20 * 40 * 60
UBA*01:01 : MKGFILLVLGIGLLHTASAVTHALKYFYTASSEVPNFPEFVVVGVVDGVQMVHYDSNSQRAVPKQD : 66
UBA*34:01 : -------------------............................................... : 47
UBA*53:01 : --------.......................................................... : 58
UBA*34:02 : --------...................................AM..................... : 58
UBA*05:01 : ---............................................................... : 63
UBA*45:01 : ---------------............................AM..................... : 58
UBA*46:01 : ---------------................................................... : 58
UBA*46:02 : ---------------..........................A.........F.............. : 58
UBA*19:02 : ---------------..........................A........................ : 58
UBA*40:01 : ---------------................................................... : 58
UBA*40:03 : -------------------......................A.........F.............. : 47
UBA*40:02 : ---------------................................................... : 58
UBA*43:01 : ---------------................................................... : 58
UBA*44:01 : ---------------................................................... : 58
UBA*18:03 : ---------------..........................A........................ : 58
UBA*04:01 : -------I..............S....................SM..................... : 59
UBA*04:03 : ---------------.......S....................SM..................... : 58
UBA*68:01 : ---------------..........................A........................ : 58
UBA*04:02 : -------------------...S....................SM..................... : 47
UBA*11:01 : --------..............S.G..................SM..................... : 58
UBA*11:02 : ---------------.......S....................SM..................... : 58
UBA*67:01 : ---------------.......S....................SM..................... : 58
UBA*22:01 : -------------------......................A........................ : 47
UBA*22:02 : -------------------......................A........................ : 47
UBA*13:01 : -------------------...S....................SM..................... : 47
UBA*37:01 : --------..............S....................SM..................... : 58
UBA*47:01 : ---------------.......S....................SM..................... : 58
UBA*21:01 : --------..............S....................SM..................... : 58
UBA*71:01 : ---------------..........................A........................ : 58
UBA*18:01 : -------------------............................................... : 47
UBA*18:02 : -------------------............................................... : 47
UBA*29:01 : -------------------...T..................A...............C........ : 47
UBA*39:01 : ---------------................................................... : 58
UBA*35:01 : -------------..................................................... : 53
UBA*35:02 : ---------------..........................A.........F.............. : 58
UBA*27:01 : -------------------......................A.........F.............. : 47
UBA*19:01 : -------------------...................G..A........................ : 47
UBA*41:01 : ---------------.T.....S..................A..M......F.............. : 58
UBA*42:01 : ---------------.T.....S..................A..M......F.............. : 58
UBA*56:01 : ---------------.T.....S..................A..M......F.............. : 58
UBA*02:01 : ---........V....V..A.NT.Q.....T.GID......TM.I.N.H.ID....ITK..IQ.AE : 63
UBA*02:02 : ---------------.V..A.NT.Q.....T.GID.....MTM.I.N.H.ID....ITK..IQ.AE : 58
UBA*20:01 : -------------------A.NT.Q.....T.GID......TM.I.N.H.ID....ITK..IQ.AE : 47
UBA*20:03 : ---------------.V..A.NT.Q.....T.GID.....MTM.I.N.H.ID....ITK..IQ.AE : 58
UBA*20:02 : ---------------------NT.Q.....T.GID......TM.I.N.H.ID....ITK..IQ.AE : 45
UBA*24:01 : -------------------A.NT.Q.....T.GIG......TM.I.N.H.ID....ITK..IQ.AE : 47
UBA*24:02 : -------------------A.NT.Q.....T.GID......TM.I.N.H.ID....ITK..IQ.AE : 47
UBA*24:05 : -------------------A.NT.Q.....T.GID.....MTM.I.N.H.ID....ITK..IQ.AE : 47
UBA*24:04 : -------------------A.NT.Q.....T.GID......TM.I.N.H.ID....ITK..IQ.AE : 47
UBA*24:03 : -------------------A.NT.Q.....T.GID......TM.I.N.H.ID....ITK..IQ.AE : 47
UBA*23:01 : -------------------A.NT.Q.....T.GID......TM.I.N.H.ID....ITK..IQ.AE : 47
UBA*63:01 : ---------------.V..A.NT.Q.....T.GID......TM.I.N.H.ID....ITK..IQ.AE : 58
UBA*49:01 : ---------------.V..A.NT.Q.....T.GID......TM.I.N.H.ID....ITK..IQ.AE : 58
UBA*03:01 : ..C....L...A-..SS..A..S.R.V...T.GI.D.....T..L.N.EPISY...IIR.ET.R.. : 65
UBA*03:02 : --C....L...A-..SS..A..S.R.V...T.GI.D.....T..L.N.EPISY...IIR.ET.R.. : 63
UBA*36:01 : --C....L...A-..SS..A..S.R.V...T.GI.D.....T..L.N.EPISY...IIR.ET.R.. : 63
UBA*36:02 : -------------------A..S.R.V...T.GI.D.....T..L.N.EPISY...IIR.ET.R.. : 54
UBA*38:01 : --C....L...A-..SS..A..S.R.V...T.GI.D.....T..L.N.EPISY...IIR.ET.R.. : 63
UBA*61:01 : -------------------A..S.R.V...T.GI.D.....T..L.N.EPISY...IIR.ET.R.. : 54
UBA*12:01 : ---------------------.S.R.V...T.GI.D.....T..L.N.EPISY...IIR.ET.R.. : 45
UBA*62:01 : -------------------A..S.R.V...T.GI.D.....T..L.N.EPISY...IIR.ET.R.. : 54
UBA*60:01 : -------------------A..S.R.V...T.GI.D.....T..L.N.EPISY...IIR.ET.R.. : 54
UBA*10:01 : PLC....L...A-..SS...I.SW.A.L...TGLSD.....ALNL..DEL.GYF.TKTN.FEG..S : 65
UBA*52:01 : -------------------.I.SW.A.L...TGLSD.....ALSLL.D...GYF.TKTN.FEG..S : 54
UBA*66:01 : -------------------.I.SW.A.L...TGLSD.....ALSLL.D...GYF.TKTN.FEG..S : 54
UBA*08:01 : ---................A..S.R.V...T.GI.D.....NL.I...M.IDY....TK....... : 63
UBA*65:01 : --------...........A..S.R.V...T.GI.D.....NL.I...M.IDY....TK....... : 58
UBA*25:01 : -------------------A..S.R.V...T.GI.D.....NL.I...M.IDY....TK....... : 47
UBA*32:01 : -------------------A..S.R.V...T.GI.D.....NL.I...M.IDY....TK....... : 47
UBA*31:01 : -------------------A..S.R.V...T.GI.D.....NL.I...M.IDY....TK....... : 47
UBA*30:01 : -------------------A..S.R.V...T.GI.D.....NL.I...M.IDY....TK....... : 47
UBA*28:01 : -------------------...S....................SM..................... : 47
UBA*54:01 : --------.................................A........................ : 58
UBA*55:01 : --------...........A..S.R.V...T.GI.D.....NL.I...M.IDY....TK....... : 58
UBA*09:01 : ----..................S.R.....TTGI.D.....D....N.KVISY...IIK.K....S : 62
UBA*06:01 : ---................A..S.......V.GDID....TI..L.NNG.F.Y....IK.M...TE : 63
UBA*06:03 : --------...........A..S.......V.GDID....TI..L.NNG.F.Y....IK.M...TE : 58
UBA*06:02 : -------------------A..S.......V.GDID....TI..L.NNG.F.Y....IK.M...TE : 47
UBA*07:01 : ---................A..S.......V.GDID....TI..L.NNG.F.Y....IK.M...TE : 63
UBA*58:01 : --------...........A..S.......V.GDID....TI..L.NNG.F.Y....IK.M...TE : 58
UBA*59:01 : --------...........A..S.......V.GDID....TI..L.NNG.F.Y....IK.M...TE : 58
UBA*14:01 : ...................A..S.......V.GDID....T...L..EG.FMYF...TKT....TE : 66
UBA*64:01 : --------...........A..S.......V.GDID....T...L..EG.FMYF...TKT....TE : 58
UBA*51:01 : --------...........A..S.......V.GDID....T...L..EG.FMYF...TKT....TE : 58
UBA*26:01 : -------------------A..S.......V.GDID....T...L..EG.FMYF...TKT....TE : 47
UBA*26:02 : -------------------A..S.......V.GDID....T...L..EG.FMYF...TKT....TE : 47
UBA*26:03 : --------...........A..S.......V.GDID....T...L..EG.FMYF...TKT....TE : 58
UBA*17:01 : -------------------AA.S.......V.GDID....T...L..EG.FMYF...TKT....TE : 47
UBA*16:01 : -------------------A..S.......V.GDID....T...L..EG.FMYF...TKT....TE : 47
UBA*15:01 : ---------.......T..AI.T..N....A.GDIG....II..L..NTPYLYF...TKT....TE : 57
UBA*50:01 : --------........T..AI.T..N....A.GDIG....II..L..NTPYLYF...TKT....TE : 58
UBA*57:01 : --------........T..A..S.......V.GDID....T...L..KG.FMYF...TKT....TE : 58
UBA*48:01 : --------...........A..S.......V.GDID....TI..L.NNG.F.Y....IK.M...TE : 58
UBA*33:01 : -----FII.LL.-INA......S......G.TGIEG..Q..A..I...MHIDYF..V.EKN.L..S : 60
UBA*33:02 : ------------------------.....G.TGIEG..Q..A..I...MHIDYF..V.EKN.L..S : 41
UBA*73:01 : ------------------------.....G.TGIEG..Q..A..I...MHIDYF..V.EKN.L..S : 50
UBA*74:01 : ------------------------.....G.TGIEG..Q..A..I...MHIDYF..V.EKN.L..S : 50
UBA*74:02 : ------------------------.....G.TGIEG..Q..A..I...MHIDYF..V.EKN.L..S : 41
UBA*75:01 : ------------------------.....G.TGIEG..Q..A..I...MHIDYF..V.EKN.L..S : 41
UBA*76:01 : ------------------------.....G.TGIEG..Q..A..I...MHIDYF..V.EKN.L..S : 41
UBA*77:01 : ------------------------.....G.TGIEG..Q..A..I...MHIDYF..V.EKN.L..S : 41
 Alpha 2 domain
 * 80 * 100 * 120
UBA*01:01 : WVNKAAD--PQYWERNTGIFKGSQQTFKANIDIAKQRFNQSG--GVHVNQWMYGCEWDDEAGVTEG : 128
UBA*34:01 : .......--.................................--...................... : 109
UBA*34:02 : .......--........................V........--...................... : 120
UBA*53:01 : .......--.................................--...TV.L.....LG.D-.I.R. : 119
UBA*05:01 : .......--.................................--......R.........T..... : 125
UBA*45:01 : .......--........................V........--...IY.N............... : 120
UBA*46:01 : .......--.........NC.....I.......V........--......N............... : 120
UBA*46:02 : .......--.........NC.....I.......V........--......N............... : 120
UBA*19:02 : .......--.........N.......................--....F.N.........T.A... : 120
UBA*40:01 : .......--.........NC.....I.......V........--....F.N.........T..... : 120
UBA*40:03 : .......--.........NC.....I.......V........--....F.N.........T..... : 109
UBA*40:02 : .......--.................................--....F.N.........T..... : 120
UBA*43:01 : .......--.................................--....F.V...........A... : 120
UBA*44:01 : .......--.................................--......K.........T..... : 120
UBA*18:03 : .......--.........N.......................--....F.M.C.........A... : 120
UBA*04:01 : .M....EAL....DIE..K.L..H.S.......C........--....F.K...........A... : 123
UBA*04:03 : .M....EAL....DIE..K.L..H.S.......C........--....F.K...........A... : 122
UBA*68:01 : .M....EAL....DIE..NLL..H.S.......C........--....F.K...........A... : 122
UBA*04:02 : CI....EAL....DIE..K.L..H.S.......C........--....F.K...........A... : 111
UBA*11:01 : .M....EAL....DIE..K.L..H.S.......C........--...IV.M.C.......T..... : 122
UBA*11:02 : .M....EAL....DIE..K.L..H.S.......C........--...IV.R.C.......T..... : 122
UBA*67:01 : .M....EAL....DIE..K.L..H.S.......C........--....F.M.C.........A... : 122
UBA*22:01 : .M....EAL....DIE..NLL..H.S.......C........--...IV.R.C.......T..... : 111
UBA*22:02 : .M....EAL....DIE..NLLA.H.S.......S........--...IV.K.........T..... : 111
UBA*13:01 : .M....EAL....DIE..K.L..H.S.......C........--...IV.K.........T..... : 111
UBA*37:01 : .M....EAL....DIE..K.L..H.S.......C........--................T..... : 122
UBA*47:01 : .M....EAL....DIE..K.L..H.S.......C........--......K.........T..... : 122
UBA*21:01 : .M....EAL....DIE..K.L..H.S.......C........--...................... : 122
UBA*71:01 : .M....EAL....DIE..NLL..H.S.......C........--...................... : 122
UBA*18:01 : .......--.................................--....F.M.C.........A... : 109
UBA*18:02 : .......--.................................--....F.M.C.........T... : 109
UBA*29:01 : .......--.........NC.....I.......V........--....F.M.C.........A... : 109
UBA*39:01 : .......--.........NC.....I.......V........--....F.K...........A... : 120
UBA*35:01 : .......--.........NC.....I.......V........--....F.N............... : 115
UBA*35:02 : .......--.........NC.....I.......V........--....F.N............... : 120
UBA*27:01 : .......--.........NC.....I.......V........--................T..... : 109
UBA*19:01 : .......--.........N.......................--....F.ICM.......T.A... : 109
UBA*41:01 : .M...TETL.........NC.....N.......VQ.......--....F.N............... : 122
UBA*42:01 : .M...TETL.........NC.....N.......VQ.......--....F.............A..V : 122
UBA*56:01 : .M...TETL.........NC.....N.......VQ.......--....V.K.........T....V : 122
UBA*02:01 : .ISG.V.--.D..KT..Q.YA.TETV.VN..NV..S....T.--......K.........T..... : 125
UBA*02:02 : .ISG.V.--.D..KT..Q.YA.TETV.VN..NV..S....T.--......K.........T..... : 120
UBA*20:01 : .ISG.V.--.D..KT..Q.YA.TETV.VN..NV..S....T.--....F.M.C.........A... : 109
UBA*20:03 : .ISG.V.--.D..KT..Q.YA.TETV.VN..NV..S....T.--....F.M.C.........A... : 120
UBA*20:02 : .ISG.V.--.DI.KT..Q.YA.TETV.VN..NV..S....T.--....F.M.C.........A... : 107
UBA*24:01 : .ISG.V.--.D..KT..Q.YA.TETV.VN..NV..S....T.--...IV.R.C.......T..... : 109
UBA*24:02 : .ISG.V.--.D..KT..Q.YA.TETV.VN..NV..S....T.--...IV.R.C.......T..... : 109
UBA*24:05 : .ISG.V.--.D..KT..Q.YA.TETV.VN..NV..S....T.--...IV.R.C.......T..... : 109
UBA*24:04 : .ISG.V.--.D..KT..Q.YA.TETV.VN..NV..S....T.--...IV.M.C.......T..... : 109
UBA*24:03 : .ISG.V.--.D..KT..Q.YA.TETV.VN..NV..S....T.--...IV.R.C.......T..... : 109
UBA*23:01 : .ISG.V.--.D..KT..Q.YA.TETV.VN..NV..S....T.--..P...K.........T..... : 109
UBA*63:01 : .ISG.V.--.D..KT..Q.YA.TETV.VN..NV..S....T.--......N............... : 120
UBA*49:01 : .ISG.V.--.D..KT..Q.YA.TETV.VN..NV..S....T.--....F.N............... : 120
UBA*03:01 : .MA.TEG--SD...SQ.QVSI..E........V.......T.--......K.........T..... : 127
UBA*03:02 : .MA.TEG--SD...SQ.QVSI..E........V.......T.--......K...S.....T..... : 125
UBA*36:01 : .MA.TEG--SD...SQ.QVSI..E........V.......T.--....F.RT.............. : 125
UBA*36:02 : .MA.TEG--SD...SQ.QVSI..E........V.......T.--....F.R............... : 116
UBA*38:01 : .MA.TEG--SD...SQ.QVSI..E........V.......T.--...................... : 125
UBA*61:01 : .MA.TEG--SD..D.Q.QVSI.DE........V.......T.--...IV.K............... : 116
UBA*12:01 : .MA.TEG--SD...SQ.QVSI..E........V.......T.--....F.M.C.........A... : 107
UBA*62:01 : .MKE.V.--.D..N...QTSI.DE........V.......T.--......R.........T..... : 116
UBA*60:01 : .MKE.V.--.D..N...QTSI.DE........V.......T.--...IY.N............... : 116
UBA*08:01 : .MA.TEG--SD..D.Q.QVSI..E.........V......T.--......N............... : 125
UBA*65:01 : .MA.TEG--SD..D.Q.QVSI..E.........V......T.--...IV.K.........T..... : 120
UBA*25:01 : .MA.TEG--SD..D.Q.QVSI..E.........V......T.--...................... : 109
UBA*32:01 : .MA.TEG--SD..D.Q.QVSI..E.........V......T.--...................... : 109
UBA*31:01 : .MA.TEG--SD..D.Q.QVSI..E.........V......T.--...................... : 109
UBA*30:01 : .MA.TEG--SD.RD.Q.QVSI..E.........V......T.--..................A..W : 109
UBA*28:01 : .M....EAL....DIE..K.L..H.S.......C........--...IF.Y....T...DS...D. : 111
UBA*54:01 : .......--.........N.......................--...IF.Y....T...DS...D. : 120
UBA*55:01 : .MA.TEG--SD..D.Q.QVSI..E.........V......T.--...IF.Y....T...DS...D. : 120
UBA*09:01 : .MEENLN--Q...NQG.DQL..TE.S.....QV.QT....T.--...IF.Y....T...DS...D. : 124
UBA*06:01 : .MKQS.G--AD..DTESEKQV.QN.G..N..QVL.D.....MST...................... : 127
UBA*06:03 : .MKQS.G--AD..DTESEKQV.QN.G..N..QVL.D.....MST....S................. : 122
UBA*06:02 : .MKQS.G--AD..DTESEKQV.QN.G..N..QVP.D.....MST...................... : 111
UBA*07:01 : .MKQS.G--AD..DTESEKQV.QN.G..N..QVL.D.....MST....F.V...........A... : 127
UBA*58:01 : .MKQS.G--AD..DTESEKQV.QN.G..N..QVL.D.....MST...IV.K.........T..... : 122
UBA*59:01 : .MKQS.G--AD..DTESEKQV.QN.G..N..QVL.D.....MST....F.K...........A... : 122
UBA*14:01 : .MK.SVG--AD..D.Q.Q.GI.AH.N.....QV..D.....KST....F.K...........A... : 130
UBA*64:01 : .MK.SVG--AD..D.Q.Q.GI.AH.N.....QV..D.....KST....F.M.C.........A... : 122
UBA*51:01 : .MK.SVG--AD..D.Q.Q.GI.AH.N.....QV..D.....KST....F.N............... : 122
UBA*26:01 : .MK.SVG--AD..D.Q.Q.GI.AH.N.....QV..D.....KST....F.N.........T..... : 111
UBA*26:02 : .MK.SVG--AD..D.Q.Q.GI.AH.N.....QV..D.....KST....F.N.........T..... : 111
UBA*26:03 : .MK.SVG--AD..D.Q.Q.GI.AH.N.....QV..D.....KST....F.N.........T..... : 122
UBA*17:01 : .MK.SVG--AD..D.Q.Q.GI.AH.N.....QV..D.....KST...................... : 111
UBA*16:01 : .MK.SVG--AD..D.Q.Q.GI.AH.N.....QV..D.....KST......N............... : 111
UBA*15:01 : .MK.SVG--AD..DSM.Q.GI.AN.I.....QVV.D.....KST....F.N............... : 121
UBA*50:01 : .MK.SVG--AD..DSM.Q.GI.AN.I.....QVV.D.....KST....F.K...........A... : 122
UBA*57:01 : .MKRE-G--AD..D.Q.Q.GI.AH....N..QV..D.....KST....F.N...........A... : 121
UBA*48:01 : .MKQS.G--AD..DTESEKQV.QN.G..N..QVL.D.....MST...IF.Y....T...DS...D. : 122
UBA*33:01 : .MEG.R.-----EKSI.N.R..H..S....VE.VM.....TT--....F.K...........A... : 119
UBA*33:02 : .MEG.R.-----EKSI.N.R..N..S....VE.VM.....TT--....F.K...........A... : 100
UBA*74:01 : .MEG.R.-----EKSI.N.R..N..S....VE.VM.....TT--...................... : 109
UBA*74:02 : .MEG.R.-----EKSI.N.R..H..S....VE.VM.....TT--...................... : 100
UBA*77:01 : .MEG.R.-----EKSI.N.R..H..S....VE.VM.....TT--................T..... : 100
UBA*73:01 : .MEG.R.-----EKSI.N.R..N..S....VE.VM.....TT--....F.N.........T..... : 109
UBA*76:01 : .MEG.R.-----EKSI.N.R..H..S....VE.VM.....TT--....F.N.........T.A... : 100
UBA*75:01 : .MEG.R.-----EKSI.N.R..H..S....VE.VM.....TT--...IF.Y....T...DS...D. : 100
UBA*10:01 : ..EEKLG--Q..L.QQEN.LRSTS.S..V.VG..ME....TK--....F.N.........T..... : 127
UBA*52:01 : ..EEKLG--Q..M.QQENLIRSTS.S..V.VG..ME....TK--......K.........T..... : 116
UBA*66:01 : ..EEKLG--Q..M.QQENLIRSTS.S..V.VG..ME....TK--....F.M.C.........A... : 116
 * 140 * 160 * 180 *
UBA*01:01 : FEQWGYDGEDFIAFDLKTKSWIAPTPQSVITKLKWDSDTAQNEHDKHYLTQTCIEWLKKYLDYGKS : 194
UBA*34:01 : ...........................A................R.N.Y..I........V.---- : 171
UBA*34:02 : ...........................A................R.N.Y..I........V..... : 186
UBA*05:01 : .N.D..............LK.......A........NNM..IQQ...................... : 191
UBA*45:01 : .D.Y.......L......LK........L.......NNM..IQQ.................----- : 181
UBA*46:01 : .D.Y.......L......LK........L.......NNM..IQQ.................----- : 181
UBA*46:02 : .D.Y.......L......LK........L.......NNM..IQQ.................----- : 181
UBA*19:02 : .N.D.......L......LK.........N..H....N..Y...W.N....E.........----- : 181
UBA*40:01 : .D.D.......L......LT.......A.N..H....N..Y..QE.N....I.........----- : 181
UBA*40:03 : .D.D.......L......LT.......A.N..H....N..Y..QE.N....I..------------ : 164
UBA*40:02 : .D.D.......L......LT.......A.N..H....N..Y..QE.N....I.........----- : 181
UBA*43:01 : .D.Y.......L......LK.......A...............YR.N.Y............----- : 181
UBA*44:01 : .D.D.......L......LT.......A.........N.....YR.N..............----- : 181
UBA*18:03 : .D.Y.......................A.........N.....YW.N....E.........----- : 181
UBA*04:01 : LT.Y...............--.......L...........Y..QE.N....I........V..... : 187
UBA*04:03 : LT.Y........................L...........Y..QE.N....I.........----- : 183
UBA*68:01 : LT.Y........................L...........Y..QE.N....I.........----- : 183
UBA*04:02 : LT.Y........................L...........Y..QE.N....I........V..... : 177
UBA*11:01 : .N.D.......................A.........N.....YW.N....E........V..... : 188
UBA*11:02 : .N.D.......................A.........N.....YW.N....E.........----- : 183
UBA*67:01 : .D.Y.......................A.........N.....YW.N....E.........----- : 183
UBA*22:01 : .N.D.......................A.........N.....YW.N....E........V..... : 177
UBA*22:02 : .N.D.......................A.........N.....YW.N....E..------------ : 166
UBA*13:01 : .N.Y..............LK.......A...............YR.N.Y...........V.---- : 173
UBA*37:01 : .N.D.......................A...............YL.N....E.............. : 188
UBA*47:01 : .D.D.......L......LT.......A.........N.....YR.N..............----- : 183
UBA*21:01 : ...........................A................R.N.Y..I........V..... : 188
UBA*71:01 : ...........................A................R.N.Y..I.........----- : 183
UBA*18:01 : .D.Y.......................A.........N.....YW.N....E........V..... : 175
UBA*18:02 : .D.Y.......................A....I....N..H..CW.N....E........V..... : 175
UBA*29:01 : .D.Y.........L.............A.........N.....YW.N....E........V..... : 175
UBA*39:01 : LT.Y........................L...........Y..QE.N....I.........----- : 181
UBA*35:01 : .D.Y...............T....KT.A.N..N..........FL.N.Y................. : 181
UBA*35:02 : .D.Y...............T....KT.A.N..N..........FL.N.Y............----- : 181
UBA*27:01 : .N.D.......................A...............YL.N....E..........---- : 171
UBA*19:01 : .N.D.......L......LK.........N..H....N..Y...W.N....E........V..... : 175
UBA*41:01 : .Y.F....K.........LK.T.....A.........NM.F..QK.N....I.........----- : 183
UBA*42:01 : LT.F..............LT.......A....N....N..L...W.N....E.........----- : 183
UBA*56:01 : LT.F.......L......LT....K..A.........N..Y..QE.N..............----- : 183
UBA*02:01 : .D.D.......L......LT.......A.........N.....YR.N................... : 191
UBA*02:02 : .D.D.......L......LT.......A.........N.....YR.N..............----- : 181
UBA*20:01 : .D.Y.......................A.........N.....YW.N....E........V.---- : 171
UBA*20:03 : .D.Y.......................A.........N.....YW.N....E.........----- : 181
UBA*20:02 : .D.Y.......................A.........N.....YW.N....E........V..... : 173
UBA*24:01 : .N.D.......................A.........N.....YW.N....E........V..... : 175
UBA*24:02 : .N.D.......................A.........N.....YW.N....E........V.---- : 171
UBA*24:05 : .N.D.......................A.........N.....YW.N....E..------------ : 164
UBA*24:04 : .N.D.......................A.........N.....YW.N....E..------------ : 164
UBA*24:03 : .N.N........Q..............A.........N.....YW.N....E........VG.... : 175
UBA*23:01 : .N.D..............LK.......A........NNM..IQQ...................... : 175
UBA*63:01 : .D.Y.......L......LK........L.......NNM..IQQ.................----- : 181
UBA*49:01 : .D.Y...............T....KT.A.N..N..........FL.N.Y............----- : 181
UBA*03:01 : .D.D.......L......LT.......A.........N.....YR.N................... : 193
UBA*03:02 : .D.D.......L......LT.......A.........N.....YR.N................E.. : 191
UBA*36:01 : ...........................A............Y..QE.N....I........V..... : 191
UBA*36:02 : ...........................A............Y..QE.N....I.........----- : 177
UBA*38:01 : ...........................A................R.N.Y..I........V..... : 191
UBA*61:01 : .N.D.......................A............Y..QK.N....I.........----- : 177
UBA*12:01 : .D.Y.......................A.........N.....YW.N....E........V..... : 173
UBA*62:01 : .N.D.......................A............Y..QK.N....I.........----- : 177
UBA*60:01 : .D.Y.......L......LK........L.......NNM..IQQ.................----- : 177
UBA*08:01 : .D.Y.......L......LK........L.......NNM..IQQ...................... : 191
UBA*65:01 : .N.Y..............LK.......A...............YR.N.Y............----- : 181
UBA*25:01 : ...........................A................R.N.Y..I...R....V..... : 175
UBA*32:01 : ........K..M...M...........A................RED.Y..M........V..... : 175
UBA*31:01 : ...Y.......................A.........N.....YW.N....E........V..... : 175
UBA*30:01 : ...........................A.........G..H..YR.N.YSHI.T..M...VE.... : 175
UBA*28:01 : LR.Y.......LVY.M.AFT....KL.AE..TR..NNEP..M.YL.S.I..E.V......V.---- : 173
UBA*54:01 : LR.Y.......LVY.M.AFT....KL.AE..TR..NNEP..M.YL.S.I..E.V.......----- : 181
UBA*55:01 : LR.Y.......LVY.M.AFT....KL.AE..TR..NNEP..M.YL.S.I..E.V.......----- : 181
UBA*09:01 : LR.Y.......LVY.M.AFT....KL.AE..TR..NNEP..M.YL.S.I..E.V......V....N : 190
UBA*06:01 : ...........................A................R.N.Y..I........V..... : 193
UBA*06:03 : ......................V....A................R.NHY..I........V..... : 188
UBA*06:02 : ...........................A................R.N.Y..I........V..... : 177
UBA*07:01 : .D.Y.......L......LK.......A...............YR.N.Y...........V..... : 193
UBA*58:01 : .N.Y..............LK.......A...............YR.N.Y............----- : 183
UBA*59:01 : LT.Y........................L...........Y..QE.N....I.........----- : 183
UBA*14:01 : LT.Y........................L...........Y..QE.N....I........V..... : 196
UBA*64:01 : .D.Y.......................A.........N.....YW.N....E.........----- : 183
UBA*51:01 : .D.Y...............T....KT.A.N..N..........FL.N.Y............----- : 183
UBA*26:01 : .D.D.......L......LT.......A.N..H....N..Y..QR.N....I........V..... : 177
UBA*26:02 : .DHD.......L......LT.......A.N..H....N..Y..QE.N....I....M...V..... : 177
UBA*26:03 : .D.D.......L......LT.......A.N..H....N..Y..QE.N....I.........----- : 183
UBA*17:01 : ...........................A................R.N.Y..I........V..... : 177
UBA*16:01 : .D.Y.......L......LK........L.......NNM..IQQ...................... : 177
UBA*15:01 : .D.Y....G.......E..T....KT.A.N..N..........FL.N.Y................. : 187
UBA*50:01 : LT.Y........................L...........Y..QE.N....I.........----- : 183
UBA*57:01 : .Y.F....K..........T....K..AF...N.L..N..H..YL.N.F..E.........----- : 182
UBA*48:01 : LR.Y.......LVY.M.AFT....KL.AE..TR..NNEP..M.YL.S.I..E.V.......----- : 183
UBA*33:01 : LT.Y........................L...........Y..QE.N....I........V..... : 185
UBA*33:02 : LT.Y........................L...........Y..QE.N....I...----------- : 155
UBA*74:01 : ...........................A................R.N.Y..I.........----- : 170
UBA*74:02 : ...........................A................R.N.Y..I...----------- : 155
UBA*77:01 : .N.D.......................A...............YL.N....E...----------- : 155
UBA*73:01 : .D.D.......L......LT.......A.N..H....N..Y..QE.N....I.........----- : 170
UBA*76:01 : .N.D.......L......LK.........N..H....N..Y...W.N....E...----------- : 155
UBA*75:01 : LR.Y.......LVY.M.AFT....KL.AE..TR..NNEP..M.YL.S.I..E.V.----------- : 155
UBA*10:01 : .D.D.......L......LT.......A.N..H....N..Y..QE.N....I........V..... : 193
UBA*52:01 : .D.D.......L......LT.......A.........N.....YR.N..............----- : 177
UBA*66:01 : .D.Y.......................A.........N.....YW.N....E.........----- : 177
UBA*53:01 : DY.F....A..LSL.KS.LT.T.ANQKA........ATG.EANFQ.D..EN..........----- : 180

Alpha 3 domain
 200 * 220 * 240 * 260
UBA*01:01 : TLMRTVPPSVSLLQKTPSSPVTCHATGFYPSGVMVSWQKDGQDHHEDVEYGETLQNDDGTFQKSSH : 260
UBA*34:01 : ------------------------------------------------------------------ : -
UBA*34:02 : .................................................................. : 252
UBA*05:01 : .................................................H................ : 257
UBA*04:01 : .................................................................. : 253
UBA*04:02 : .....------------------------------------------------------------- : 182
UBA*11:01 : .................................................H................ : 254
UBA*22:01 : ....-------------------------------------------------------------- : 181
UBA*37:01 : ......................................................P.........I. : 254
UBA*21:01 : .................................................................. : 254
UBA*18:01 : ....-------------------------------------------------------------- : 179
UBA*18:02 : ....-------------------------------------------------------------- : 179
UBA*29:01 : ....-------------------------------------------------------------- : 179
UBA*35:01 : .................................................................. : 247
UBA*19:01 : ....-------------------------------------------------------------- : 179
UBA*02:01 : .................................................H................ : 257
UBA*20:02 : .................................................H................ : 239
UBA*24:01 : ....-------------------------------------------------------------- : 179
UBA*24:03 : ....-------------------------------------------------------------- : 179
UBA*23:01 : ....-------------------------------------------------------------- : 179
UBA*03:01 : .................................................H................ : 259
UBA*03:02 : .................................................H................ : 257
UBA*36:01 : .................................................................. : 257
UBA*38:01 : .................................................................. : 257
UBA*12:01 : .................................................H................ : 239
UBA*08:01 : .................................................H................ : 257
UBA*25:01 : ....-------------------------------------------------------------- : 179
UBA*32:01 : ....-------------------------------------------------------------- : 179
UBA*31:01 : ....-------------------------------------------------------------- : 179
UBA*30:01 : .V..-------------------------------------------------------------- : 179
UBA*09:01 : ...............S...................F.......Q..........P.H......... : 256
UBA*06:01 : .................................................................. : 259
UBA*06:03 : .................................................................. : 254
UBA*06:02 : .....------------------------------------------------------------- : 182
UBA*07:01 : .................................................H................ : 259
UBA*14:01 : .................................................................. : 262
UBA*26:01 : ....-------------------------------------------------------------- : 181
UBA*26:02 : ....-------------------------------------------------------------- : 181
UBA*17:01 : ....-------------------------------------------------------------- : 181
UBA*16:01 : ....-------------------------------------------------------------- : 181
UBA*15:01 : .................................................H................ : 253
UBA*33:01 : .................................................................. : 251
UBA*10:01 : .................................................................. : 259
 CP/TM/CYT domains
 * 280 * 300 * 320
UBA*01:01 : LTVTPEEWKNNKYQCVVQVTGVKEDFIKVLTESEIKTNWNEPNIVLIIVVVVALLLLVVAV--VVG : 324
UBA*34:02 : ........................................D....................--... : 316
UBA*05:01 : .....................LQ.................D....................--... : 321
UBA*04:01 : ........................................D....................--... : 317
UBA*11:01 : .....................I.D.I..............D.......G...........A--.A. : 318
UBA*37:01 : ......DR...........K.I.........D------LDD.......G.......V....----- : 309
UBA*21:01 : ........................................D....................--... : 318
UBA*35:01 : .................H......................D..............P.....--... : 311
UBA*02:01 : .....................LQ.................D.......G.......V....--... : 321
UBA*20:02 : .....................I.D.I..............D.......G...........A--.A. : 303
UBA*03:01 : .....................LQ.................D.......G.......V....--... : 323
UBA*03:02 : .....................LQ.................D.......G..E....V....--... : 321
UBA*36:01 : ........................................D....................--... : 321
UBA*38:01 : ........................................D....................--... : 321
UBA*12:01 : .....................I.D.I..............D.......G...........A--.A. : 303
UBA*08:01 : .....................LQ.................D....................--... : 321
UBA*09:01 : ......DR.............I.D.......DLDDP.----....P..........V....--... : 316
UBA*06:01 : ........................................D....................--... : 323
UBA*06:03 : ........................................D....................--... : 318
UBA*07:01 : .....................I.D.I..............D.......G...........A--.A. : 323
UBA*14:01 : ........................................D....................--... : 326
UBA*15:01 : ...................K.LQ.................D....................--... : 317
UBA*33:01 : ........................................D....................--... : 315
UBA*10:01 : .....................LQ.................D....................--... : 323

 * 340 *
UBA*01:01 : VVIWKKKSKKGFVPASTSDTDSDNSGRAAQMT : 356
UBA*34:02 : .............................L.. : 348
UBA*05:01 : ................................ : 353
UBA*04:01 : ................................ : 349
UBA*11:01 : ................................ : 350
UBA*21:01 : ......-------------------------- : 324
UBA*35:01 : .......................H.------- : 336
UBA*02:01 : ................................ : 353
UBA*20:02 : ................................ : 335
UBA*03:01 : ................................ : 355
UBA*03:02 : ......-------------------------- : 327
UBA*36:01 : ......-------------------------- : 327
UBA*38:01 : ......-------------------------- : 327
UBA*12:01 : ................................ : 335
UBA*08:01 : ................................ : 353
UBA*09:01 : ......................E...KG..KI : 348
UBA*06:01 : ................................ : 355
UBA*06:03 : ................................ : 350
UBA*07:01 : ................................ : 355
UBA*14:01 : ................................ : 358
UBA*15:01 : ...............................A : 349
UBA*33:01 : ................................ : 347
UBA*10:01 : .............................L.. : 355

**Alignment of deduced *DAB* amino acid sequences**

The longer version of *DAB*09:01* is shown using _L.

Leader sequence Beta 1 domain
 * 20 * 40 *
DAB*01:01 : ---------------MSIFCVSLTLVLSIFSGT DGYFEQVVRQCRYSSKDLQGIEFIDSYVFNKAEYI : 53
DAB*01:02 : -------------MS.................. ......................L............ : 55
DAB*02:01 : -------------MS.................. ..................................V : 55
DAB*02:02 : --------------------............. .....H............................V : 48
DAB*03:01 : -------------MS.................. ....YHMM..............L.T.....Q.... : 55
DAB*03:03 : -------------MS.................. ....YHMM..............L.T.....Q.... : 55
DAB*03:02 : --------------------------------- ....YHMM..............L.T.....Q.... : 35
DAB*06:01 : -------------MS.................. ....YHMMT.............L.T.....Q..N. : 55
DAB*04:01 : -------------MS.................. ....F...................H.....Q..N. : 55
DAB*05:01 : -------------MS.................. ....Y.............................V : 55
DAB*07:01 : -------------MS.................. ..................................V : 55
DAB*08:01 : -------------MS.................. ....Y.R.SE........................V : 55
DAB*08:02 : --------------------............. ....Y.R.SE........................V : 48
DAB*09:01 : --------------------------------- ..................................V : 35
DAB*09:01_L: -------------MS.................. ..................................V : 55
DAB*09:02 : --------------------............. ..................................V : 48
DAB*10:01 : --------------------............. ....H.R.T.....................Q.... : 48
DAB*11:01 : --------------------............. ....H.R.T.....................Q.... : 48
DAB*11:02 : --------------------------------- ....H.R.T.....................Q.... : 35
DAB*12:01 : --------------------............. ................................... : 48
DAB*12:02 : --------------------------------- ................................... : 35
DAB*13:01 : --------------------------------- ....YHMM..............L.T.....Q..N. : 35
DAB*13:02 : --------------------------------- ....YHMM..............L.T.....Q..N. : 35
DAB*13:03 : -------------MS.................. ....YHMM..............L.T.....Q..N. : 55
DAB*14:01 : --------------------------------- ....Y.........................Q.... : 35
DAB*15:01 : --------------------------------- ....Y.........................Q.... : 35
DAB*15:02 : -------------MS.................. ....Y.........................Q.... : 55
DAB*15:03 : -------------MS.................. ....Y.........................Q.... : 55
DAB*16:01 : --------------------------------- ..............................Q.... : 35
DAB*17:01 : --------------------------------- ............F.......T.............V : 35
DAB*17:02 : --------------------------------- ........S...F.......T.............V : 35
DAB*17:03 : -------------MS.................. ........S...F.......T.............V : 55
DAB*18:01 : --------------------------------- ....H.R.T.....................Q.... : 35
DAB*19:01 : --------------------------------- ....YHMM..............L.T.....Q.... : 35
DAB*20:01 : -------------MS.................. ....F...................H.....Q..N. : 55
DAB*20:02 : -------------MS.................. ....F...................H.....Q..N. : 55
DAB*21:01 : -------------MS.................. ....Y.R.SE....................Q..N. : 55
DAB*22:01 : --------------------------------- ....YHMM..............L.T.....Q..N. : 35
DAB*22:02 : -------------MS.................. ....YHMM..............L.T.....Q..N. : 55
DAB*22:03 : -------------MS.................. ....YHMM..............L.T.....Q..N. : 55
DAB*23:01 : --------------------------------- ....H.R.T.....................Q.... : 35
DAB*23:02 : -------------MS.................. ....H.R.T..P..................Q.... : 55
DAB*24:01 : -------------MS.................. ....F.........................Q..N. : 55
DAB*25:01 : --------------------------------- ........S...F...........H.......... : 35
DAB*26:01 : -------------MS.................. ........S...F...........H.......... : 55
DAB*27:01 : -------------MS.................. ....YHMM..............L.T.....Q..N. : 55
DAB*28:01 : -------------MS.................. ........S...F...........H.......... : 55

Beta 2 domain
 60 * 80 * 100 * 120
DAB*01:01 : RFNSTVGKFVGYTELGVKNAEAWNSDAAV-LAVERGELERYCKHNADLHYSTILDKT VEPHVRLSSVA : 120
DAB*01:02 : .............................-........................... ........... : 122
DAB*02:01 : ........Y...............-KGPE-....L.....F.....AIY..A..... ........... : 121
DAB*02:02 : ........Y...............-KGPE-....L.....F.....AIY..A..... ........... : 114
DAB*03:01 : ........Y.....Y.........-KGPE-..G.L.....V.....PID..A..... ........... : 121
DAB*03:03 : ........Y.....Y.........-KGPE-..G.L.V.........PIY..A..... ........... : 121
DAB*03:02 : ........Y.....Y.........-KGPE-..G.L.....V.....PIY..------ ----------- : 84
DAB*06:01 : ..............H.........-KGPE-..G.L.V......F..PID..A..... ........... : 121
DAB*04:01 : ........Y...............-KGPE-..G.L.....F................ ........... : 121
DAB*05:01 : ........Y.....Y.........-KGPE-..G.L...........PIY..A..... ........... : 121
DAB*07:01 : ........Y...............-KGPE-....L.....F.........RA..... ........... : 121
DAB*08:01 : ........Y.....Y.........-KGPE-..G.L.....V.....PID..A..... ........... : 121
DAB*08:02 : ........Y.....Y.........-KGPE-..G.L.....V.....PIY..A..... ........... : 114
DAB*09:01 : ........Y...............-KGPE-....L........L..PID..------ ----------- : 84
DAB*09:01_L: ........Y...............-KGPE-....L........L..PID..A..... ........... : 121
DAB*09:02 : ........................-KGPE-....L........L..PID..A..... ........... : 114
DAB*10:01 : ......................L.-KGPE-....L........L..PID..A..... ........... : 114
DAB*11:01 : ..............H.........-KGPE-..G.L........F..PIY..A..... ........... : 114
DAB*11:02 : ..............H.........-KGPE-..G.L.....V.....PIY..------ ----------- : 84
DAB*12:01 : ........Y.....Y.........-KGPE-..RAL.....V.....PIY..A..... ........... : 114
DAB*12:02 : ........Y.....Y.........-KGPE-..RAL........F..PIY..------ ----------- : 84
DAB*13:01 : ........................-KGPE-..G.L.V...F..........------ ----------- : 84
DAB*13:02 : ..............H.........-KGPE-..G.L.....F..........------ ----------- : 84
DAB*13:03 : ..............H.........-KGPE-..G.L.....V................ ........... : 121
DAB*14:01 : ..............H.........-KGPE-....L................------ ----------- : 84
DAB*15:01 : ..............H.........-KGPE-....L.....F.....PID..------ ----------- : 84
DAB*15:02 : ..............H.........-KGPE-..G.L........L..PID..A..... ........... : 121
DAB*15:03 : ..............H.........-KGPE-....L........L..PID..A..... ........... : 121
DAB*16:01 : ........................-KGPE-....L........L..PID..------ ----------- : 84
DAB*17:01 : ........Y...............-KGPE-..G.L.....F.....PID..------ ----------- : 84
DAB*17:02 : ........Y...............-KGPE-....L.....F..Q..PID..------ ----------- : 84
DAB*17:03 : ........Y...............-KGPE-....L.....F.....PID..A..... ........... : 121
DAB*18:01 : ..............H.........-SD.AG..G.L.....F.........------- ----------- : 84
DAB*19:01 : ........Y.....Y.........-KGPE-..G.L...........AN...------ ----------- : 84
DAB*20:01 : ........Y...............-KGPE-....L............ID..A..... ........... : 121
DAB*20:02 : ........Y...............-KGPE-....L.V..........ID..A..... ........... : 121
DAB*21:01 : ........................-KGPE-..G.L.....F................ ........... : 121
DAB*22:01 : ..............H.........-KGPE-..G.L.....F.....PID..------ ----------- : 84
DAB*22:02 : ........................-KGPE-..G.L.....V.....PID..A..... ........... : 121
DAB*22:03 : ..............H.........-KGPE-..G.L.....V.....PIY..A..... ........... : 121
DAB*23:01 : ........................-KGPE-..G.L.V..........ID..------ ----------- : 84
DAB*23:02 : ........................-KGPE-..G.L.V..........ID..A..... ........... : 121
DAB*24:01 : ........................-KGPE-..RAL........L..PIY..A..... ........... : 121
DAB*25:01 : ........Y...............-KGPE-..G.L.....F.....PID..------ ----------- : 84
DAB*26:01 : ........Y...............-KGPE-..G.L.....F................ ........... : 121
DAB*27:01 : ........................-KGPE-..G.L.V......L..PIY........ ........... : 121
DAB*28:01 : ........Y...............-KGPE-..G.L........L..PID..A..... ........... : 121

 * 140 * 160 * 180
DAB*01:01 : PPSGRHPAMLMCSAYDFYPKPIRVTWLRDGREVKSDVTSTEELANGDWYYQIHSHLEYTPRSGEKISCM : 189
DAB*01:02 : ..................................................................... : 191
DAB*02:01 : ..................................................................... : 190
DAB*02:02 : ..................................................................... : 183
DAB*03:01 : .................F................................................... : 190
DAB*03:03 : .................F................................................... : 190
DAB*06:01 : .................F................................................... : 190
DAB*04:01 : ..................................................................... : 190
DAB*05:01 : ..........I.......................................................... : 190
DAB*07:01 : ..................................................................... : 190
DAB*08:01 : .................F................................................... : 190
DAB*08:02 : .................F................................................... : 183
DAB*09:01_L: .................................................-------------------- : 170
DAB*09:02 : ..................................................................... : 183
DAB*10:01 : ..................................................................... : 183
DAB*11:01 : ..................................................................... : 183
DAB*12:01 : ..................................................................... : 183
DAB*13:03 : .................F................................................... : 190
DAB*15:02 : ..................................................................... : 190
DAB*15:03 : .................................................-------------------- : 170
DAB*17:03 : ..................................................................... : 190
DAB*20:01 : ..................................................................... : 190
DAB*20:02 : .................................................-------------------- : 170
DAB*21:01 : ..................................................................... : 190
DAB*22:02 : .................F...............................-------------------- : 170
DAB*22:03 : .................F...............................-------------------- : 170
DAB*23:01 : --------------------------------------------------------------------- : -
DAB*23:02 : ..................................................................... : 190
DAB*24:01 : ..................................................................... : 190
DAB*25:01 : --------------------------------------------------------------------- : -
DAB*26:01 : ..................................................................... : 190
DAB*27:01 : .................F................................................... : 190
DAB*28:01 : ..................................................................... : 190

CP/ TM/ CYT domains
 * 200 * 220 * 240
DAB*01:01 : VEHISLTEPMVYHW DPSLPEAERNKIAIGASGLVLGAILALAGLIYYKKKSSGVL------------ : 244
DAB*01:02 : .............. ..................----------------------------------- : 223
DAB*02:01 : .............. ..................----------------------------------- : 222
DAB*02:02 : .............. ...........------------------------------------------ : 208
DAB*03:01 : .............. ..................----------------------------------- : 222
DAB*03:03 : .............. ..................----------------------------------- : 222
DAB*06:01 : .............. .........................................------------ : 245
DAB*04:01 : .............. ..................----------------------------------- : 222
DAB*05:01 : .............. ..................----------------------------------- : 222
DAB*07:01 : .............. .........................................------------ : 245
DAB*08:01 : .............. ..................----------------------------------- : 222
DAB*08:02 : .............. ...........------------------------------------------ : 208
DAB*09:02 : .............. ...........------------------------------------------ : 208
DAB*10:01 : .............. ...........------------------------------------------ : 208
DAB*11:01 : .............. ...........------------------------------------------ : 208
DAB*12:01 : .............. ...........------------------------------------------ : 208
DAB*13:03 : .............. ..................----------------------------------- : 222
DAB*15:02 : .............. ..................----------------------------------- : 222
DAB*17:03 : .............. ..................----------------------------------- : 222
DAB*20:01 : .............. ..................----------------------------------- : 222
DAB*21:01 : .............. ..................----------------------------------- : 222
DAB*23:02 : .............. ..................----------------------------------- : 222
DAB*24:01 : .............. ..................----------------------------------- : 222
DAB*26:01 : .............. ..................----------------------------------- : 222
DAB*27:01 : .............. ..................----------------------------------- : 222
DAB*28:01 : .............. ..................----------------------------------- : 222

**Alignment of deduced *DAA* amino acid sequences**

Signal sequence Alpha 1 domain
 Forward primer

* 20 * 40 * 60 *
DAA*01:01 : MKTSVIVLILCWQVYAEHKVLHIDLVITGCSDSDGLDMYGLDGEEMWYADFNKQEGVVALPPFADPFTFP : 70
DAA*01:02 : ----------............................................................ : 60
DAA*08:01 : --------------------.....A............................................ : 50
DAA*07:01 : ----------...............A..........................................YH : 60
DAA*07:02 : -----------------........A..........................................YH : 60
DAA*02:01 : ----------...............Y.S.........................G................ : 60
DAA*04:01 : ----------...............H.I.......V.................G................ : 60
DAA*09:01 : ------.............................V.................G...............H : 64
DAA*09:03 : -----------------........A.........V.................G...............H : 54
DAA*09:02 : -----------------..................V.................G...............H : 60
DAA*03:01 : ----------.........................V.................G...MP.........Y. : 60
DAA*03:02 : --------------------.....A...........................G...MP.........Y. : 50
DAA*03:03:01: --------------------................N................G...MP.........Y. : 50
DAA*03:03:02: --------------------................N................G...MP.........Y. : 50
DAA*03:04 : ----------------------------.......V.................G...MP.........Y. : 42
DAA*05:01 : ----------...............A.........V.................G................ : 60
DAA*05:02 : ----------...............A.........V.................G................ : 60
DAA*11:02 : ----------------.........H.........V.................G................ : 54
DAA*10:01_ : ------...................A..........N................G................ : 64
DAA*11:01_ : --------------------.....A.........V..........S......G................ : 50
DAA*14:01_ : ----------............K..Y...........................G................ : 60
DAA*06:01 : ----------.................S.......V.................G...MP.........Y. : 60
DAA*13:01 : ----------.................S.......V.................G................ : 60
DAA*12:01 : --------------------.....H.........VE................G...MP.........Y. : 50
DAA*12:02 : ----------------------------.......VE................G...MP........... : 42
DAA*03:05 : ----------------------------........N................G...MP.........Y. : 42
DAA*08:02 : -----------------........Y.S.......................................... : 54
DAA*16:01 : -----------------........Y.S.......VN..............I.G...LP.........Y. : 60
DAA*17:01 : -----------------.....K..Y.M.......................I.G.........V..LS.. : 60
DAA*15:01 : -----------------.......FW.S.......V.........L.....I.G.........V..LS.. : 60
DAA*18:01 : -----------------.......VH.........V.........K.....I.G....VQ.......NY. : 60

 Alpha 2 domain
 80 * 100 * 120 * 140
DAA*01:01 : GFYEQAVGNQGVCKGNLAKCIKAYKNPEEKIDPPHSSIYPRDDVDLGVENTLICHVSGFFPAPVRVRWTR : 140
DAA*01:02 : ...................................................................... : 130
DAA*08:01 : .H..G......................................................H.......... : 120
DAA*07:01 : .A.........I...............................................H........A. : 130
DAA*07:02 : .A.........I...............................................H.......... : 130
DAA*02:01 : ....G.........A...VN.......................................H.......... : 130
DAA*04:01 : ....G.........A...VN.................................................. : 130
DAA*09:01 : .A..G.........A...VN.......................................H.......... : 134
DAA*09:03 : .A..G.........A...VN.......................................H.......... : 124
DAA*09:02 : .A..G.........A...VN........................V..............H.......... : 130
DAA*03:01 : .A..G......I..A...T............A...........................H.......... : 130
DAA*03:02 : .A..G......I..A...T............A...........................H.......... : 120
DAA*03:03:01: .A..G......I..A...T............A...........................H.......... : 120
DAA*03:03:02: .A..G......I..A...T............--------------------------------------- : 81
DAA*03:04 : .A............A...T..........----------------------------------------- : 71
DAA*05:01 : .H..G.........A...TS...........A...........................Y.......... : 130
DAA*05:02 : ..................TS...........A...........................Y.......... : 130
DAA*11:02 : ..................TS...........A...........................H.......... : 124
DAA*10:01 : ..............A...TS...........A...........................H.......... : 134
DAA*11:01 : ..............A...TS...........A...........................H.......... : 120
DAA*14:01 : ..............A...T............A...........................H.......... : 130
DAA*06:01 : .A............A...VN...........A...........................Y.......... : 130
DAA*13:01 : .A............A...VN...........A...................................... : 130
DAA*12:01 : .A.........I..A...VN.......................................H.......... : 120
DAA*12:02 : .A............A...VN.........----------------------------------------- : 71
DAA*03:05 : .A..R.........A...I..........----------------------------------------- : 71
DAA*08:02 : .H..G......................................................H..S....... : 124
DAA*16:01 : .A......EL....A...TS...........A............E..............Y.......... : 130
DAA*17:01 : .A............A..DVA.......................................H.......... : 130
DAA*15:01 : ........Q.....A...TA.......P.T.A...................................... : 130
DAA*18:01 : .A......QH....A...VD.......P.T.............................H......M... : 130

Reverse primer CP/TM/CYT domains
 * 160 * 180 * 200 *
DAA*01:01 : NNQNLTEGVRLSTPYPNADFTLNQFSSLPFTPEEGDIYGCTVEHKGLAEPLTRIWEPEVIQPSVGPDVFC : 210
DAA*01:02 : ..................................................................A... : 200
DAA*08:01 : ..................................................................A... : 190
DAA*07:01 : ..................................................................A... : 200
DAA*07:02 : ..............-------------------------------------------------------- : 151
DAA*02:01 : ..................................................................A... : 200
DAA*04:01 : ..................................................................A... : 200
DAA*09:01 : ..................................................................A... : 204
DAA*09:03_ : ..............-------------------------------------------------------- : 139
DAA*09:02 : ..............-------------------------------------------------------- : 151
DAA*03:01 : ..................................................................A... : 200
DAA*03:02 : ..................................................................A... : 190
DAA*03:03:01: ..................................................................A... : 190
DAA*05:01 : ...................V.F............................................A... : 200
DAA*05:02 : ...................V.F............................................A... : 200
DAA*11:02 : ..............-------------------------------------------------------- : 139
DAA*10:01 : ..................................................................A... : 204
DAA*11:01 : ..................................................................A... : 190
DAA*14:01 : ..................................................................A... : 200
DAA*06:01 : ...................V.F............................................A... : 200
DAA*13:01 : ...................V.F............................................A... : 200
DAA*12:01 : ..................................................................A... : 190
DAA*08:02 : ..............-------------------------------------------------------- : 139
DAA*16:01 : ..............-------------------------------------------------------- : 151
DAA*17:01 : ..............-------------------------------------------------------- : 151
DAA*15:01 : ..............-------------------------------------------------------- : 151
DAA*18:01 : ..............-------------------------------------------------------- : 151

220 *
DAA*01:01 : GVGLTLGLLGVAAGTFFLIKGNQCN : 235
DAA*01:02 : ......................... : 225
DAA*08:01 : ......................... : 215
DAA*07:01 : ......................... : 225
DAA*02:01 : .....V................... : 225
DAA*04:01 : .....V................... : 225
DAA*09:01 : .....V................... : 229
DAA*03:01 : ......................... : 225
DAA*03:02 : ......................... : 215
DAA*03:03:01: ......................... : 215
DAA*05:01 : .....V................... : 225
DAA*05:02 : .....V................... : 225
DAA*10:01 : ......................... : 229
DAA*11:01 : ......................... : 215
DAA*14:01 : ......................... : 225
DAA*06:01 : ......................... : 225
DAA*13:01 : ......................... : 225
DAA*12:01 : .....V................... : 215

**SF1.3. MHC allele frequencies and *DAA*-*DAB* haplotypes**

***UBA* allele frequencies in each population**

|  |  | Etne | Granvin | Jondal | Kinso | Opo | Rosendal | Steinsdal | Ådland | Farmed | Total |
| --- | --- | --- | --- | --- | --- | --- | --- | --- | --- | --- | --- |
| 1 | UBA*02:01 | 13.8 | 9.1 | 8.8 | 4.8 | 2.9 | 6.3 | 3.9 | 8.9 | 5.1 | 6.68 |
| 2 | UBA*02:02 | 1.3 | 2.3 | 0 | 0 | 0 | 0 | 0 | 0 | 0 | 0.28 |
| 3 | UBA*03:01 | 0 | 4.6 | 0 | 4.8 | 14.4 | 3.8 | 7.7 | 1.1 | 5.7 | 5.26 |
| 4 | UBA*04:03 | 0 | 4.6 | 0 | 4.8 | 1.0 | 1.3 | 0 | 2.2 | 1.1 | 1.42 |
| 5 | UBA*05:01 | 1.3 | 0 | 5.9 | 2.4 | 1.0 | 0 | 3.9 | 0 | 2.8 | 1.71 |
| 6 | UBA*06:01 | 6.3 | 9.1 | 0 | 9.5 | 7.7 | 6.3 | 7.7 | 4.4 | 7.4 | 6.68 |
| 7 | UBA*07:01 | 3.8 | 15.9 | 11.8 | 4.8 | 4.8 | 1.3 | 1.9 | 3.3 | 6.3 | 5.26 |
| 8 | UBA*08:01 | 6.3 | 0 | 5.9 | 7.1 | 7.7 | 1.3 | 3.9 | 0 | 7.4 | 4.83 |
| 9 | UBA*09:01 | 0 | 2.3 | 0 | 0 | 0 | 1.3 | 1.9 | 0 | 0 | 0.43 |
| 10 | UBA*10:01 | 1.3 | 0 | 5.9 | 2.4 | 9.6 | 2.5 | 5.8 | 2.2 | 3.4 | 3.84 |
| 11 | UBA*11:02 | 0 | 0 | 5.9 | 0 | 0 | 0 | 1.9 | 2.2 | 0 | 0.71 |
| 12 | UBA*13:01 | 8.8 | 6.8 | 11.8 | 11.9 | 8.7 | 13.8 | 3.9 | 22.2 | 6.8 | 10.37 |
| 13 | UBA*14:01 | 0 | 0 | 2.9 | 2.4 | 0 | 0 | 1.9 | 0 | 2.3 | 0.99 |
| 14 | UBA*18:03 | 0 | 2.3 | 0 | 0 | 1.0 | 1.3 | 3.9 | 3.3 | 2.8 | 1.85 |
| 15 | UBA*19:02 | 0 | 2.3 | 2.9 | 0 | 0 | 0 | 0 | 0 | 0 | 0.28 |
| 16 | UBA*20:01 | 8.8 | 18.2 | 5.9 | 4.8 | 6.7 | 6.3 | 9.6 | 5.6 | 1.1 | 6.11 |
| 17 | UBA*20:03 | 0 | 0 | 2.9 | 0 | 1.0 | 5.0 | 0 | 0 | 0 | 0.85 |
| 18 | UBA*21:01 | 6.3 | 0 | 2.9 | 0 | 0 | 5.0 | 0 | 2.2 | 1.7 | 2.13 |
| 19 | UBA*22:02 | 0 | 0 | 0 | 0 | 1.0 | 0 | 0 | 0 | 0 | 0.14 |
| 20 | UBA*24:02 | 3.8 | 0 | 2.9 | 2.4 | 0 | 2.5 | 3.9 | 4.4 | 0.6 | 1.99 |
| 21 | UBA*24:04 | 1.3 | 0 | 0 | 0 | 0 | 0 | 0 | 0 | 0 | 0.14 |
| 22 | UBA*24:05 | 0 | 0 | 0 | 0 | 1.0 | 0 | 0 | 0 | 0 | 0.14 |
| 23 | UBA*26:03 | 0 | 0 | 5.9 | 0 | 1 | 0 | 0 | 5.6 | 0 | 1.14 |
| 24 | UBA*27:01 | 3.8 | 0 | 0 | 0 | 0 | 0 | 1.9 | 0 | 0 | 0.57 |
| 25 | UBA*28:01 | 0 | 0 | 0 | 2.4 | 0 | 0 | 0 | 0 | 0 | 0.14 |
| 26 | UBA*33:01 | 1.3 | 0 | 0 | 0 | 2.9 | 0 | 0 | 3.3 | 0.6 | 1.14 |
| 27 | UBA*33:02 | 1.3 | 2.3 | 0 | 0 | 5.8 | 1.3 | 1.9 | 0 | 0 | 1.42 |
| 28 | UBA*34:01 | 7.5 | 0 | 2.9 | 2.4 | 0 | 8.8 | 7.7 | 1.1 | 3.4 | 3.69 |
| 29 | UBA*34:02 | 0 | 4.6 | 0 | 2.4 | 1.0 | 0 | 0 | 1.1 | 0.6 | 0.85 |
| 30 | UBA*35:01 | 0 | 0 | 0 | 0 | 0 | 0 | 0 | 0 | 0.6 | 0.14 |
| 31 | UBA*35:02 | 0 | 0 | 0 | 4.8 | 1.0 | 0 | 1.9 | 0 | 1.1 | 0.85 |
| 32 | UBA*36:02 | 1.3 | 0 | 5.9 | 2.4 | 1.9 | 1.3 | 1.9 | 2.2 | 11.9 | 4.40 |
| 33 | UBA*37:01 | 1.3 | 0 | 0 | 0 | 0 | 3.8 | 0 | 0 | 14.2 | 4.12 |
| 34 | UBA*38:01 | 0 | 4.6 | 0 | 0 | 0 | 0 | 0 | 2.2 | 1.1 | 0.71 |
| 35 | UBA*39:01 | 2.5 | 0 | 0 | 4.8 | 0 | 0 | 0 | 2.2 | 0 | 0.85 |
| 36 | UBA*40:01 | 0 | 0 | 0 | 4.8 | 0 | 0 | 1.9 | 2.2 | 4.6 | 1.85 |
| 37 | UBA*40:02 | 0 | 0 | 0 | 0 | 1.0 | 1.3 | 0 | 0 | 0 | 0.28 |
| 38 | UBA*40:03 | 0 | 0 | 0 | 0 | 0 | 0 | 0 | 0 | 0.6 | 0.14 |
| 39 | UBA*42:01 | 0 | 0 | 0 | 0 | 0 | 0 | 1.9 | 0 | 0 | 0.14 |
| 40 | UBA*43:01 | 2.5 | 0 | 0 | 0 | 0 | 3.8 | 0 | 0 | 0 | 0.71 |
| 41 | UBA*44:01 | 0 | 0 | 5.9 | 0 | 0 | 0 | 0 | 0 | 0 | 0.28 |
| 42 | UBA*45:01 | 0 | 0 | 0 | 7.1 | 0 | 3.8 | 1.9 | 0 | 1.1 | 1.28 |
| 43 | UBA*46:01 | 0 | 0 | 0 | 0 | 1.0 | 3.8 | 0 | 6.7 | 0 | 1.42 |
| 44 | UBA*46:02 | 0 | 0 | 0 | 2.4 | 0 | 0 | 0 | 0 | 0 | 0.14 |
| 45 | UBA*47:01 | 0 | 4.6 | 0 | 0 | 0 | 0 | 0 | 0 | 0 | 0.28 |
| 46 | UBA*48:01 | 1.3 | 0 | 0 | 0 | 0 | 0 | 0 | 4.4 | 0 | 0.71 |
| 47 | UBA*49:01 | 1.3 | 0 | 0 | 0 | 0 | 0 | 0 | 1.1 | 0 | 0.28 |
| 48 | UBA*50:01 | 0 | 4.6 | 2.9 | 0 | 0 | 1.3 | 0 | 0 | 0 | 0.57 |
| 49 | UBA*51:01 | 0 | 0 | 0 | 0 | 0 | 1.3 | 0 | 0 | 0 | 0.14 |
| 50 | UBA*52:01 | 0 | 2.3 | 0 | 0 | 0 | 1.3 | 0 | 2.2 | 0 | 0.57 |
| 51 | UBA*53:01 | 0 | 0 | 0 | 0 | 0 | 1.3 | 0 | 0 | 0 | 0.14 |
| 52 | UBA*54:01 | 0 | 0 | 0 | 4.8 | 0 | 1.3 | 0 | 0 | 0 | 0.43 |
| 53 | UBA*55:01 | 1.3 | 0 | 0 | 0 | 0 | 1.3 | 0 | 1.1 | 0 | 0.43 |
| 54 | UBA*56:01 | 0 | 0 | 0 | 0 | 1.9 | 0 | 0 | 0 | 0 | 0.28 |
| 55 | UBA*57:01 | 0 | 0 | 0 | 0 | 0 | 0 | 1.9 | 0 | 0 | 0.14 |
| 56 | UBA*58:01 | 0 | 0 | 0 | 0 | 0 | 0 | 3.9 | 0 | 0.6 | 0.43 |
| 57 | UBA*59:01 | 0 | 0 | 0 | 0 | 0 | 0 | 1.9 | 0 | 0 | 0.14 |
| 58 | UBA*60:01 | 1.3 | 2.3 | 0 | 0 | 2.9 | 0 | 1.9 | 0 | 0 | 0.85 |
| 59 | UBA*61:01 | 1.3 | 0 | 0 | 0 | 4.8 | 0 | 0 | 0 | 0 | 0.85 |
| 60 | UBA*62:01 | 0 | 0 | 0 | 0 | 0 | 0 | 1.9 | 0 | 0 | 0.14 |
| 61 | UBA*63:01 | 1.2 | 0 | 0 | 0 | 0 | 0 | 1.9 | 0 | 0 | 0.28 |
| 62 | UBA*64:01 | 1.2 | 0 | 0 | 0 | 0 | 0 | 0 | 0 | 0 | 0.14 |
| 63 | UBA*65:01 | 0 | 0 | 0 | 0 | 1.0 | 0 | 0 | 0 | 0 | 0.14 |
| 64 | UBA*66:01 | 0 | 0 | 0 | 0 | 2.9 | 0 | 0 | 0 | 0 | 0.43 |
| 65 | UBA*67:01 | 0 | 0 | 0 | 0 | 0 | 5.0 | 0 | 0 | 0 | 0.57 |
| 66 | UBA*68:01 | 0 | 0 | 0 | 0 | 1.9 | 0 | 0 | 0 | 0 | 0.28 |
| 67 | UBA*71:01 | 0 | 0 | 0 | 0 | 0 | 0 | 0 | 0 | 0.6 | 0.14 |
| 68 | UBA*74:01 | 1.3 | 0 | 0 | 0 | 0 | 0 | 3.9 | 0 | 2.3 | 0.99 |
| 69 | UBA*74:02 | 0 | 0 | 0 | 0 | 1.0 | 0 | 0 | 0 | 0 | 0.14 |
| 70 | UBA*75:01 | 5.0 | 0 | 0 | 0 | 0 | 1.3 | 0 | 1.1 | 0.6 | 0.99 |
| 71 | UBA*76:01 | 0 | 0 | 0 | 0 | 0 | 0 | 0 | 1.1 | 0 | 0.14 |
| 72 | UBA*77:01 | 1.3 | 0 | 0 | 0 | 0 | 1.3 | 0 | 0 | 1.7 | 0.71 |
|  | Total # alleles | 30 | 18 | 18 | 22 | 29 | 31 | 29 | 27 | 30 |  |
|  | # Fish | 40 | 22 | 17 | 21 | 52 | 40 | 26 | 45 | 90 |  |
|  | Population | Etne | Granvin | Jondal | Kinso | Opo | Rosendal | Steinsdal | Ådland | Farmed |  |

Frequencies shown in percentage. Alleles found in single populations are shown using blue shading.

***DAB* allele frequencies in each population**

|  |  | Etne | Granvin | Jondal | Kinso | Opo | Rosendal | Steinsdal | Ådland | Farmed |
| --- | --- | --- | --- | --- | --- | --- | --- | --- | --- | --- |
| 1 | DAB*01:01 | 3.7 | 0 | 0 | 0 | 0 | 0 | 1.9 | 0 | 1.7 |
| 2 | DAB*02:01 | 17.1 | 11.3 | 8.8 | 21.4 | 10.2 | 8.8 | 13.5 | 11.1 | 20.5 |
| 3 | DAB*02:02 | 0 | 0 | 0 | 0 | 1.0 | 1.3 | 0 | 2.2 | 2.3 |
| 4 | DAB*03:01 | 4.9 | 9.1 | 14.7 | 7.1 | 12.2 | 10.0 | 19.2 | 4.4 | 10.8 |
| 5 | DAB*04:01 | 2.4 | 0 | 2.9 | 0 | 1.0 | 0 | 0 | 2.2 | 2.3 |
| 6 | DAB*05:01 | 0 | 9.1 | 0 | 4.8 | 7.1 | 3.8 | 7.7 | 3.3 | 2.9 |
| 7 | DAB*06:01 | 15.9 | 13.6 | 0 | 2.4 | 15.3 | 6.3 | 3.9 | 13.3 | 2.3 |
| 8 | DAB*07:01 | 17.1 | 13.6 | 16.7 | 26.2 | 12.2 | 20.0 | 17.3 | 8.9 | 23.9 |
| 9 | DAB*08:01 | 11.0 | 18.2 | 16.7 | 14.3 | 19.4 | 12.5 | 13.5 | 7.8 | 18.2 |
| 10 | DAB*08:02 | 0 | 0 | 2.9 | 0 | 0 | 0 | 0 | 0 | 0 |
| 11 | DAB*09:01 | 6.1 | 0 | 2.9 | 0 | 1.0 | 3.8 | 1.9 | 4.4 | 2.3 |
| 12 | DAB*09:02 | 6.1 | 2.3 | 5.9 | 9.5 | 3.1 | 8.8 | 3.9 | 8.9 | 2.9 |
| 13 | DAB*10:01 | 1.2 | 2.3 | 0 | 2.4 | 1.0 | 1.3 | 0 | 3.3 | 0 |
| 14 | DAB*11:01 | 2.4 | 0 | 2.9 | 2.4 | 2.0 | 1.3 | 0 | 2.2 | 0 |
| 15 | DAB*12:01 | 0 | 2.3 | 0 | 4.8 | 2.0 | 0 | 0 | 4.4 | 2.9 |
| 16 | DAB*12:02 | 0 | 0 | 0 | 0 | 0 | 0 | 0 | 0 | 0.5 |
| 17 | DAB*15:03 | 0 | 0 | 0 | 0 | 1.0 | 0 | 0 | 0 | 0 |
| 18 | DAB*17:03 | 0 | 2.3 | 0 | 2.4 | 1.0 | 1.3 | 1.9 | 1.1 | 0 |
| 19 | DAB*20:01 | 7.3 | 13.6 | 14.7 | 0 | 7.1 | 13.8 | 9.6 | 18.9 | 5.1 |
| 20 | DAB*20:02 | 4.9 | 0 | 0 | 0 | 3.1 | 7.5 | 3.9 | 3.3 | 1.1 |
| 21 | DAB*22:02 | 0 | 2.3 | 0 | 0 | 0 | 0 | 0 | 0 | 0 |
| 22 | DAB*22:03 | 0 | 0 | 2.9 | 0 | 0 | 0 | 0 | 0 | 0.5 |
| 23 | DAB*27:01 | 0 | 0 | 0 | 2.4 | 0 | 0 | 1.9 | 0 | 0 |
|  | Total # alleles | 13 | 12 | 11 | 12 | 17 | 14 | 13 | 16 | 16 |
|  | # Fish | 41 | 22 | 17 | 21 | 49 | 40 | 26 | 45 | 90 |
|  |  | Etne | Granvin | Jondal | Kinso | Opo | Rosendal | Steinsdal | Ådland | Farmed |

Frequencies are given in percentage. Alleles found in single populations are shown using blue shading.

***DAA* allele frequencies in each population**

|  |  | Etne | Granvin | Jondal | Kinso | Opo | Rosendal | Steinsdal | Ådland | Farmed |
| --- | --- | --- | --- | --- | --- | --- | --- | --- | --- | --- |
| 1 | *DAA*01:0x* | 11.3 | 19.1 | 23.5 | 14.3 | 19.6 | 12.5 | 11.5 | 9.2 | 17.6 |
| 2 | *DAA*02:01* | 8.8 | 9.5 | 8.8 | 14.3 | 6.9 | 9.7 | 9.6 | 9.2 | 22.7 |
| 3 | *DAA*03:01* | 2.5 | 0 | 0 | 0 | 2.0 | 0 | 0 | 2.6 | 3.4 |
| 4 | *DAA*03:02* | 12.5 | 11.9 | 14.7 | 0 | 9.8 | 20.8 | 13.5 | 23.7 | 6.3 |
| 5 | *DAA*03:03:01* | 0 | 0 | 2.9 | 0 | 0 | 0 | 0 | 0 | 0 |
| 6 | *DAA*04:01* | 11.3 | 2.4 | 8.8 | 9.5 | 3.9 | 12.5 | 5.8 | 11.8 | 4.0 |
| 7 | *DAA*05:01* | 5.0 | 11.9 | 14.7 | 7.1 | 11.8 | 11.1 | 17.3 | 4.0 | 10.8 |
| 8 | *DAA*06:01* | 16.3 | 14.3 | 2.9 | 2.4 | 14.7 | 4.2 | 3.9 | 13.2 | 2.8 |
| 9 | *DAA*07:02* | 0 | 9.5 | 0 | 4.8 | 6.9 | 2.8 | 7.7 | 4.0 | 2.8 |
| 10 | *DAA*08:01* | 0 | 2.4 | 0 | 4.8 | 2.0 | 0 | 0 | 4.0 | 2.3 |
| 11 | *DAA*08:02* | 0 | 0 | 0 | 0 | 0 | 0 | 0 | 0 | 0.6 |
| 12 | *DAA*09:01* | 25.0 | 16.7 | 20.6 | 35.7 | 16.7 | 23.6 | 21.2 | 13.2 | 24.4 |
| 13 | *DAA*09:02* | 0 | 0 | 0 | 0 | 0 | 0 | 1.9 | 0 | 0 |
| 14 | *DAA*09:03* | 0 | 0 | 0 | 0 | 0 | 0 | 0 | 0 | 0.6 |
| 15 | *DAA*10:01* | 1.3 | 2.4 | 0 | 2.4 | 1.0 | 1.4 | 0 | 2.6 | 0 |
| 16 | *DAA*11:01* | 2.5 | 0 | 2.9 | 2.4 | 2.0 | 1.4 | 0 | 2.6 | 0 |
| 17 | *DAA*11:02* | 0 | 0 | 0 | 0 | 1.0 | 0 | 0 | 0 | 0 |
| 18 | *DAA*12:01* | 3.8 | 0 | 0 | 0 | 0 | 0 | 1.9 | 0 | 1.7 |
| 19 | *DAA*13:01* | 0 | 0 | 0 | 2.4 | 0 | 0 | 1.9 | 0 | 0 |
| 20 | *DAA*15:01* | 0 | 0 | 0 | 0 | 1.0 | 0 | 0 | 0 | 0 |
| 21 | *DAA*16:01* | 0 | 0 | 0 | 0 | 1.0 | 0 | 0 | 0 | 0 |
| 22 | *DAA*17:01* | 0 | 0 | 0 | 0 | 0 | 0 | 1.9 | 0 | 0 |
| 23 | *DAA*18:01* | 0 | 0 | 0 | 0 | 0 | 0 | 1.9 | 0 | 0 |
|  | Total # alleles | 11 | 10 | 9 | 11 | 15 | 10 | 13 | 12 | 13 |
|  | # Fish | 40 | 21 | 17 | 21 | 51 | 36 | 26 | 38 | 90 |
|  |  | Etne | Granvin | Jondal | Kinso | Opo | Rosendal | Steinsdal | Ådland | Farmed |

Frequencies are given in percentage. Alleles found in single populations are shown using blue shading. *DAA*01:0X* is used as we cannot discriminate between the *DAA*01:01* and *DAA*01:02* alleles in our amplified region.

***DAA*-*DAB* allele haplotypes in wild and farmed animals**

|  | Haplotype 1 | Haplotype 2 | Haplotype 3 |
| --- | --- | --- | --- |
| 1 | *DAB*01:01*-*DAA*12:01* (4)[3] |  |  |
| 2 | *DAB*02:01*-*DAA*02:01^a^*^) b)^ (32)[33] | *DAB*02:01*-*DAA*09:01* (19)[3] |  |
| 3 | *DAB*02:02*-*DAA*02:01* (1)[4] |  |  |
| 4 | *DAB*03:01*-*DAA*05:01* ^a)^ (40)[19] |  |  |
| 5 | *DAB*04:01*-*DAA*03:01* ^a)^ (5)[4] | DAB*04:01-DAA*03:03:01 (1) |  |
| 6 | *DAB*05:01*-*DAA*07:02* (16)[5] |  |  |
| 7 | *DAB*06:01*-*DAA*06:01* ^a) b)^ (45)[6] |  |  |
| 8 | *DAB*07:01*-*DAA*09:01^b^*^)^ (61)[40] | *DAB*07:01*-*DAA*09:02* (1) | *DAB*07:01*-*DAA*09:03* [1] |
| 9 | *DAB*08:01*-*DAA*01:0x* (58)[31] | *DAB*08:02*-*DAA*01:0x* (1) |  |
| 11 | *DAB*09:01*-*DAA*04:01* ^b)^ (10)[3] | *DAB*09:01*-*DAA*09:01* (4) | *DAB*09:01*-*DAA*03:01* [1] |
| 12 | *DAB*09:02*-*DAA*04:01* (25)[4] |  |  |
| 13 | *DAB*10:01*-*DAA*10:01* (4) |  |  |
| 14 | *DAB*11:01*-*DAA*11:01* (7) |  |  |
| 15 | *DAB*12:01*-*DAA*08:01* (5)[4] | *DAB*12:02*-*DAA*08:02* [1] |  |
| 16 | *DAB*12:02*-*DAA*08:02* [1] |  |  |
| 17 | *DAB*15:03*-*DAA*19:01* (1) |  |  |
| 18 | *DAB*17:03*-*DAA*02:01* (4) |  |  |
| 20 | *DAB*20:01*-*DAA*03:02* ^b)^ (36)[9] | *DAB*20:02*-*DAA*03:02* (15)[2] |  |
| 21 | DAB*22:02-DAA*06:01 (1) |  |  |
| 22 | *DAB*22:03*-*DAA*06:01* (1)[1] |  |  |
| 23 | *DAB*27:01*-*DAA*13:01* (2) |  |  |
| ND | DAB*?- *DAA*15:01*/*DAA*16:01* |  |  |

Number of haplotypes in wild animals are shown with (x) while similar in farmed animals are shown using [x]. ND defines no data. ^a)^ defines haplotypes previously identified in Stet et al.2002. ^b)^ defines haplotypes found in Sundaram et al.2020. We could not amplify the *DAB* sequence in the animal OPO19 so unknown haplotype for the *DAA*15:01* and *DAA*16:01* alleles.

**SF1.4 Sequence phylogenies**

**Evolutionary analysis by Maximum Likelihood method**

*UBA*, *DAA* and *DAB* phylogenies are based on nucleotide sequences. The percentage of trees in which the associated taxa clustered together are shown next to the branches. Initial trees for the heuristic search were obtained automatically by applying Neighbor-Join and BioNJ algorithms to a matrix of pairwise distances estimated using the Maximum Composite Likelihood (MCL) approach, and then selecting the topology with superior log likelihood value. The trees are drawn to scale, with branch lengths measured in the number of substitutions per site. Codon positions included were 1st+2nd+3rd+Noncoding. All positions with less than 95% site coverage were eliminated, i.e., fewer than 5% alignment gaps, missing data, and ambiguous bases were allowed at any position (partial deletion option). Evolutionary analyses were conducted in MEGA X (Kumar et al., 2018).

**Legend to *UBA* phylogeny:** The evolutionary history was inferred by using the Maximum Likelihood method and General Time Reversible model (Nei & Kumar 2000). The tree with the highest log likelihood (-9110,97) is shown. A discrete Gamma distribution was used to model evolutionary rate differences among sites [5 categories (+*G*, parameter = 0,4456)]. This analysis involved 97 nucleotide sequences. There were 512 positions in the final dataset.

**Legend to *DAB* phylogeny:** The evolutionary history was inferred by using the Maximum Likelihood method and Kimura 2-parameter model (Kimura 1980). The tree with the highest log likelihood (-1812,36) is shown. A discrete Gamma distribution was used to model evolutionary rate differences among sites [5 categories (+*G*, parameter = 0,5253)]. The rate variation model allowed for some sites to be evolutionarily invariable ([+*I*], 70,20% sites). This analysis involved 35 nucleotide sequences. There were a total of 488 positions in the final dataset.

**Legend to *DAA* phylogeny**: The evolutionary history was inferred by using the Maximum Likelihood method based on the Jukes-Cantor model (Jukes and Cantor 1969). The tree with the highest log likelihood (-953,45) is shown. A discrete Gamma distribution was used to model evolutionary rate differences among sites (5 categories (+*G*, parameter = 0,0924)). The analysis involved 36 nucleotide sequences. There were a total of 214 positions in the final dataset.

**References:**

Kimura M. (**1980**). A simple method for estimating evolutionary rate of base substitutions through comparative studies of nucleotide sequences. *Journal of Molecular Evolution* **16**:111-120.

Kumar S., Stecher G., Li M., Knyaz C., and Tamura K. (**2018**). MEGA X: Molecular Evolutionary Genetics Analysis across computing platforms. *Molecular Biology and Evolution* **35**:1547-1549.

Nei M. and Kumar S. (**2000**). *Molecular Evolution and Phylogenetics*. Oxford University Press, New York.

Jukes T.H. and Cantor C.R. (**1969**). Evolution of protein molecules. In Munro HN, editor, Mammalian Protein Metabolism, pp. 21-132, Academic Press, New York.


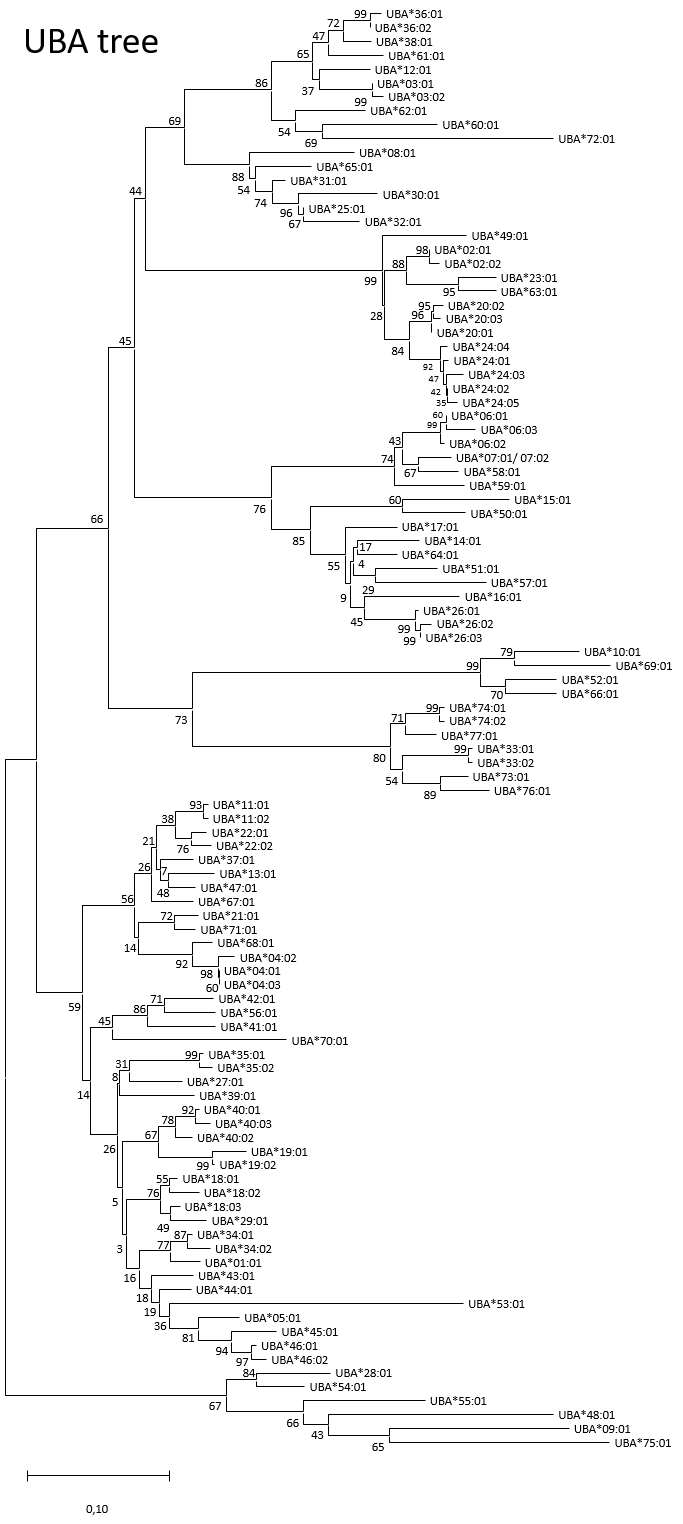


**
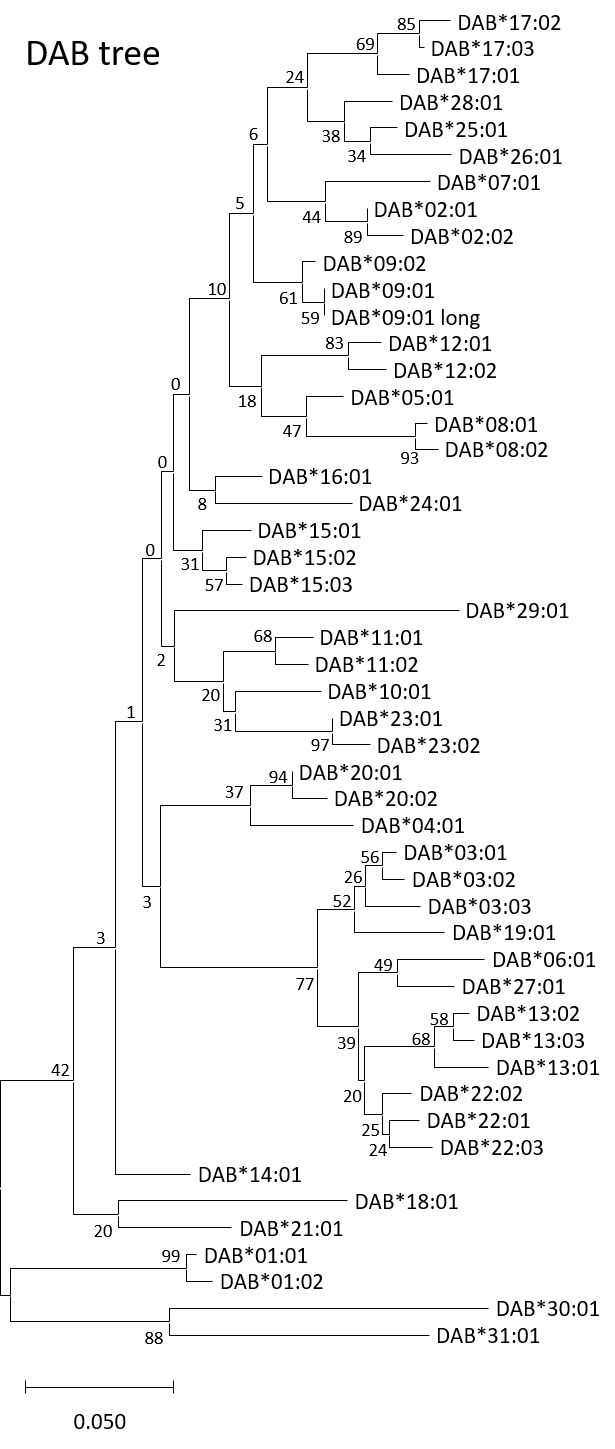
**

**
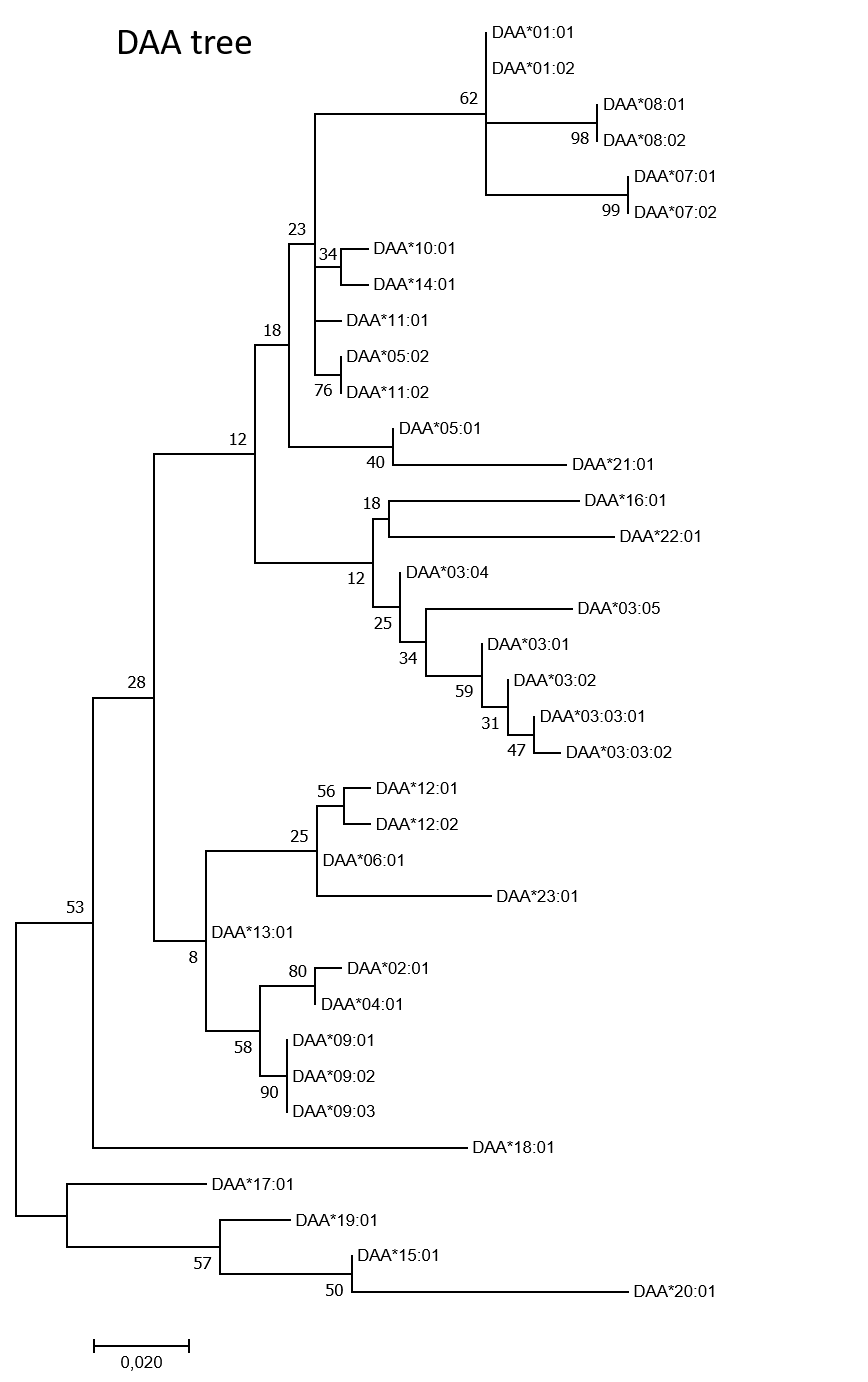
**

**SF1.5. Accession numbers for the new MHC alleles**

Accession numbers for new *UBA* alleles:

>Sasa-UBA*02:02 NCBI accession # OL441523

>Sasa-UBA*04:03 NCBI accession # OL441524

>Sasa-UBA*11:02 NCBI accession # OL441525

>Sasa-UBA*18:03 NCBI accession # OL441526

>Sasa-UBA*19:02 NCBI accession # OL441527

>Sasa-UBA*20:03 NCBI accession # OL441528

>Sasa-UBA*22:02 NCBI accession # OL441529

>Sasa-UBA*24:04 NCBI accession # OL441530

>Sasa-UBA*24:05 NCBI accession # OL441531

>Sasa-UBA*26:03 NCBI accession # OL441532

>Sasa-UBA*33:02 NCBI accession # OR667822

>Sasa-UBA*35:02 NCBI accession # OL441533

>Sasa-UBA*36:02 NCBI accession # OL441534

>Sasa-UBA*39:01 NCBI accession # OL441535

>Sasa-UBA*40:01 NCBI accession # OL441536

>Sasa-UBA*40:02 NCBI accession # OL441537

>Sasa-UBA*40:03 NCBI accession # OL441538

>Sasa-UBA*41:01 NCBI accession # OL441539

>Sasa-UBA*42:01 NCBI accession # OL441540

>Sasa-UBA*43:01 NCBI accession # OL441541

>Sasa-UBA*44:01 NCBI accession # OL441542

>Sasa-UBA*45:01 NCBI accession # OL441543

>Sasa-UBA*46:01 NCBI accession # OL441544

>Sasa-UBA*46:02 NCBI accession # OL441545

>Sasa-UBA*47:01 NCBI accession # OL441546

>Sasa-UBA*48:01 NCBI accession # OL441547

>Sasa-UBA*49:01 NCBI accession # OL441548

>Sasa-UBA*50:01 NCBI accession # OL441549

>Sasa-UBA*51:01 NCBI accession # OL441550

>Sasa-UBA*52:01 NCBI accession # OL441551

>Sasa-UBA*53:01 NCBI accession # OL441552

>Sasa-UBA*54:01 NCBI accession # OL441553

>Sasa-UBA*55:01 NCBI accession # OL441554

>Sasa-UBA*56:01 NCBI accession # OL441555

>Sasa-UBA*57:01 NCBI accession # OL441556

>Sasa-UBA*58:01 NCBI accession # OL441557

>Sasa-UBA*59:01 NCBI accession # OL441558

>Sasa-UBA*60:01 NCBI accession # OL441559

>Sasa-UBA*61:01 NCBI accession # OL441560

>Sasa-UBA*62:01 NCBI accession # OL441561

>Sasa-UBA*63:01 NCBI accession # OL441562

>Sasa-UBA*64:01 NCBI accession # OL441563

>Sasa-UBA*65:01 NCBI accession # OL441564

>Sasa-UBA*66:01 NCBI accession # OL441565

>Sasa-UBA*67:01 NCBI accession # OL441566

>Sasa-UBA*68:01 NCBI accession # OL441567

>Sasa-UBA*71:01 NCBI accession # OL441570

>Sasa-UBA*73:01 NCBI accession # OR667820

>Sasa-UBA*74:01 NCBI accession # OR667821

>Sasa-UBA*74:02 NCBI accession # OR667823

>Sasa-UBA*75:01 NCBI accession # OR667824

>Sasa-UBA*76:01 NCBI accession # OR667825

>Sasa-UBA*77:01 NCBI accession # OR667826

Accession numbers for new *DAB* alleles:

>Sasa-DAB*09:01_L NCBI accession # OL441572

>Sasa-DAB*15:03 NCBI accession # OL441573

>Sasa-DAB*20:02 NCBI accession # OL441574

>Sasa-DAB*22:02 NCBI accession # OL441575

>Sasa-DAB*22:03 NCBI accession # OL441576

**Accession numbers for new *DAA* alleles**

>Sasa-DAA*07:02 NCBI accession # OL441580

>Sasa-DAA*09:02 NCBI accession # OL441582

>Sasa-DAA*09:03 NCBI accession # OL441583

>Sasa-DAA*15:01 NCBI accession # OL441585

>Sasa-DAA*16:01 NCBI accession # OL441586

>Sasa-DAA*17:01 NCBI accession # OL441587

>Sasa-DAA*18:01 NCBI accession # OL441588

**SF1.6. *UBA* homozygosity**

Number of homozygous animals for given *UBA* alleles per each population

|  | UBA allele\Pop | Ste | Opo | Jon | Kin | Gra | Adl | Ros | Etne | Farm | Total | α1 lineage | Frequency |
| --- | --- | --- | --- | --- | --- | --- | --- | --- | --- | --- | --- | --- | --- |
| 1 | *UBA*02:01* |  |  |  |  | 1 | 1 |  | 1 | 2 | 5 | VI | 6.68 |
| 2 | *UBA*03:01* | 1 | 3 |  |  |  |  |  |  |  | 4 | III | 5.26 |
| 3 | *UBA*05:01* | 1 |  | 1 |  |  |  |  |  | 1 | 3 | I | 1.71 |
| 4 | *UBA*06:01* | 1 |  |  | 2 | 1 |  | 2 |  | 2 | 8 | V | 6.68 |
| 5 | *UBA*07:01* |  |  |  |  | 3 | 1 |  |  | 2 | 6 | V | 5,26 |
| 6 | *UBA*08:01* |  | 1 |  |  |  |  |  |  | 3 | 4 | III | 4.83 |
| 7 | *UBA*10:01* |  | 1 |  |  |  |  |  |  | 1 | 2 | VII | 3.84 |
| 8 | *UBA*11:02* |  |  | 1 |  |  | 1 |  |  |  | 2 | I | 0.71 |
| 9 | *UBA*13:01* |  | 2 | 2 | 1 |  | 6 | 1 | 1 | 2 | 15 | I | 10.37 |
| 10 | *UBA*14:01* |  |  |  |  |  |  |  |  | 1 | 1 | V | 0.99 |
| 11 | *UBA*18:03* |  |  |  |  |  |  |  |  | 1 | 1 | I | 1.85 |
| 12 | *UBA*20:01* |  | 1 |  |  | 2 |  |  | 1 |  | 4 | VI | 6.11 |
| 13 | *UBA*20:03* |  |  |  |  |  |  | 1 |  |  | 1 | VI | 0.85 |
| 14 | *UBA*21:01* |  |  |  |  |  |  | 1 | 1 |  | 2 | I | 2.13 |
| 15 | *UBA*24:02* |  |  |  |  |  | 1 |  |  |  | 1 | VI | 1.99 |
| 16 | *UBA*26:03* |  |  | 1 |  |  |  |  |  |  | 1 | V | 1.14 |
| 17 | *UBA*33:02* |  | 2 |  |  |  |  |  |  |  | 2 | IV | 1.14 |
| 18 | *UBA*36:02* |  |  |  |  |  |  |  |  | 3 | 3 | III | 4.40 |
| 19 | *UBA*37:01* |  |  |  |  |  |  | 1 |  | 6 | 7 | I | 4.12 |
| 20 | *UBA*38:01* |  |  |  |  |  |  |  |  | 1 | 1 | III | 0.71 |
| 21 | *UBA*39:01* |  |  |  | 1 |  |  |  |  |  | 1 | III | 0.85 |
| 22 | *UBA*40:01* |  |  |  |  |  |  |  |  | 3 | 3 | I | 1.85 |
| 23 | *UBA*44:01* |  |  | 1 |  |  |  |  |  |  | 1 | I | 0.28 |
| 24 | *UBA*45:01* |  |  |  | 1 |  |  |  |  |  | 1 | I | 1.28 |
| 25 | *UBA*47:01* |  |  |  |  | 1 |  |  |  |  | 1 | I | 0.28 |
| 26 | *UBA*48:01* |  |  |  |  |  | 1 |  |  |  | 1 | V | 0.71 |
| 27 | *UBA*54:01* |  |  |  | 1 |  |  |  |  |  | 1 | I | 0.43 |
| 28 | *UBA*56:01* |  | 1 |  |  |  |  |  |  |  | 1 | I | 0.28 |
| 29 | *UBA*66:01* |  | 1 |  |  |  |  |  |  |  | 1 | VII | 0.43 |
| 30 | *UBA*67:01* |  |  |  |  |  |  | 1 |  |  | 1 | I | 0.57 |
| 31 | *UBA*68:01* |  | 1 |  |  |  |  |  |  |  | 1 | I | 0.28 |
| 32 | *UBA*74:01* | 1 |  |  |  |  |  |  |  |  | 1 | IV | 0.99 |
| 33 | *UBA*77:01* |  |  |  |  |  |  |  |  | 1 | 1 | IV | 0.71 |
|  | Total | 4 | 13 | 6 | 6 | 8 | 11 | 7 | 4 | 29 |  |  |  |
|  | # Animals | 27 | 52 | 17 | 21 | 22 | 45 | 40 | 40 | 90 |  |  |  |
|  | % | 15 | 25 | 35,3 | 28,6 | 36,4 | 24,4 | 17,5 | 10 | 32 |  |  |  |
|  | UBA\Pop | Ste | Opo | Jon | Kin | Gra | Adl | Ros | Etne | Farm | Total | α1 lineage lineage | Frequency |

Population abbreviations are as follows: Ste is Steinsdal, Jon is Jondal, Kin is Kinso, Gra is Granvin, Adl is Ådland, Ros is Rosendal, Farm is farmed escapees.

**SF1.7. FST across eight salmon populations in Hardangerfjord area**

| **Locus** | **F_ST_ (Range)** |
| --- | --- |
| MHC DAB | 0.007 (0 - 0.037) |
| MHC UBA | 0.009 (0 - 0.028) |
| 68 Neutral | 0.006 (0 - 0.014) |
| Vgll3 | 0.064 (0 - 0.193) |
| Six6 | 0.094 (0 - 0.256) |

Fst values given for the MHCII DAB locus, MHCI UBA locus, 68 putatively neutral loci, Vgll3 locus, and the Six6 locus with range in FST between pairs of populations in parenthesis.
